# Itraconazole targets cell cycle heterogeneity in colorectal cancer

**DOI:** 10.1084/jem.20171385

**Published:** 2018-07-02

**Authors:** Simon J.A. Buczacki, Semiramis Popova, Emma Biggs, Chrysa Koukorava, Jon Buzzelli, Louis Vermeulen, Lee Hazelwood, Hayley Francies, Mathew J. Garnett, Douglas J. Winton

**Affiliations:** 1 Cancer Research UK (CRUK) Cambridge Institute, Li Ka Shing Centre, Robinson Way, Cambridge, England, UK; 2 Laboratory for Experimental Oncology and Radiobiology (LEXOR), Center for Experimental Molecular Medicine (CEMM), Academic Medical Center (AMC), University of Amsterdam, Amsterdam, Netherlands; 3 Cancer Research UK/Medical Research Council Oxford Institute for Radiation Oncology (OIRO), Department of Oncology, University of Oxford, Oxford, UK; 4 Wellcome Trust Sanger Institute, Wellcome Trust Genome Campus, Hinxton, England, UK

## Abstract

Cellular dormancy and heterogeneity in cell cycle length provide important explanations for treatment failure after adjuvant therapy with S-phase cytotoxics in colorectal cancer (CRC), yet the molecular control of the dormant versus cycling state remains unknown. We sought to understand the molecular features of dormant CRC cells to facilitate rationale identification of compounds to target both dormant and cycling tumor cells. Unexpectedly, we demonstrate that dormant CRC cells are differentiated, yet retain clonogenic capacity. Mouse organoid drug screening identifies that itraconazole generates spheroid collapse and loss of dormancy. Human CRC cell dormancy and tumor growth can also be perturbed by itraconazole, which is found to inhibit Wnt signaling through noncanonical hedgehog signaling. Preclinical validation shows itraconazole to be effective in multiple assays through Wnt inhibition, causing both cycling and dormant cells to switch to global senescence. These data provide preclinical evidence to support an early phase trial of itraconazole in CRC.

## Abstract (precis)

Chemotherapy-resistant dormant colorectal cancer cells are a differentiated cell that can de-differentiate and proliferate, accounting for tumor recurrence. Itraconazole induces all tumor cells, including those dormant, to proliferate then enter senescence and is effective in multiple preclinical assays.

## Introduction

Colorectal cancer (CRC) is the third most common cancer in the Western World. CRC is a heterogeneous disease and recent large scale molecular studies have identified clinically relevant overlapping subgroups that can be identified in primary tumors, primary cultures, xenografts, and traditional cell lines (De Sousa E Melo et al., 2013; Guinney et al., 2015; Linnekamp et al., 2018). This intertumoral heterogeneity is a major explanation for differential chemotherapy responses and clinical progression. Although recent advances in oncological treatment have generated marked improvements for patients with CRC, many who receive adjuvant therapy ultimately die as a result of relapse with systemic disease. There are several explanations for tumor recurrence, including cellular dormancy or quiescence that allow cancer cells to persist and reenter the cell cycle after a latent period or therapy-induced stimulation. Across cancer types, cellular dormancy has been shown to represent an important hallmark of cancer cells that facilitates immune evasion and avoidance of targeted death by S-phase cytotoxics (Kreso et al., 2013; Malladi et al., 2016). From a functional perspective, dormant CRC cells have been found to be rare, chemoresistant, and yet highly clonogenic, features compatible with a stem cell–like phenotype (Moore et al., 2012; Kreso et al., 2013). However, their true molecular identity and the mechanisms underlying dormancy remain elusive, and there is an urgent need to identify compounds that can perturb this dormant state to enable more complete cancer cell killing to prevent late recurrence.

In the normal intestine there are two stem cell populations: one rapidly dividing and another quiescent reserve population that becomes activated during tissue injury (Clevers, 2013). It is increasingly recognized that premalignant adenomas and malignant tumors contain many similar cell types as that found in the tissue of origin (Verga Falzacappa et al., 2012). Two very recent studies have identified and characterized cancer stem cell (CSC) populations in CRC (De Sousa E Melo et al., 2017; Shimokawa et al., 2017). In one study, De Sousa E Melo et al. demonstrate that liver metastases arising from primary colon cancers are highly dependent on *Lgr5*-expressing CSCs, and elimination of these cells dramatically decreases both the formation and maintenance of liver metastases, whereas in primary tumors they are redundant (De Sousa E Melo et al., 2017). In an alternative study, Shimokawa et al. (2017) show two distinct cell populations exist in human CRCs: a differentiated cell population with limited self-renewal capacity expressing *Keratin 20* (Krt20) and a proliferative CSC population expressing *Lgr5*. Intriguingly, targeting of Lgr5^+^ CSCs leads to dedifferentiation of the Krt20 population to an Lgr5^+^ status, sustaining continued tumor growth. Whether both populations are present throughout all molecular subtypes of CRC and what signaling pathways control these divergent behaviors is unknown. However, it is clear, to achieve therapeutic benefit, Lgr5^+^ CSCs and less proliferative Krt20^+^ cells must both be targeted.

The Wnt pathway is commonly hyperactivated in CRC and plays a pivotal role in intestinal stem cell maintenance. High Wnt activity has been shown to also define CSCs (Vermeulen et al., 2010). Although much is now understood about how Wnt signaling functions and several experimental compounds have been tested, it has been impossible to develop a clinically useful drug that can target the pathway. In addition to Wnt, there are several other autocrine- and paracrine-signaling pathways that are involved in controlling intestinal stem cell behavior/kinetics during epithelial homeostasis and regeneration (Beumer and Clevers, 2016). Further, there is significant cross talk between many of these signaling pathways. Perturbation of individual pathways has been shown to alter the behavior of stem cells, progenitor populations, and the proportions of differentiated cells generated. Here, we seek to characterize dormant cells in different CRC subtypes to identify controlling pathways. We hypothesize that disrupting these pathways could cause departure from dormancy with subsequent proliferation, thus rendering the tumor as a whole more susceptible to conventional therapies that target dividing cells.

## Results

### Human spheroid label–retaining cells are a differentiated population

**Figure 1.**
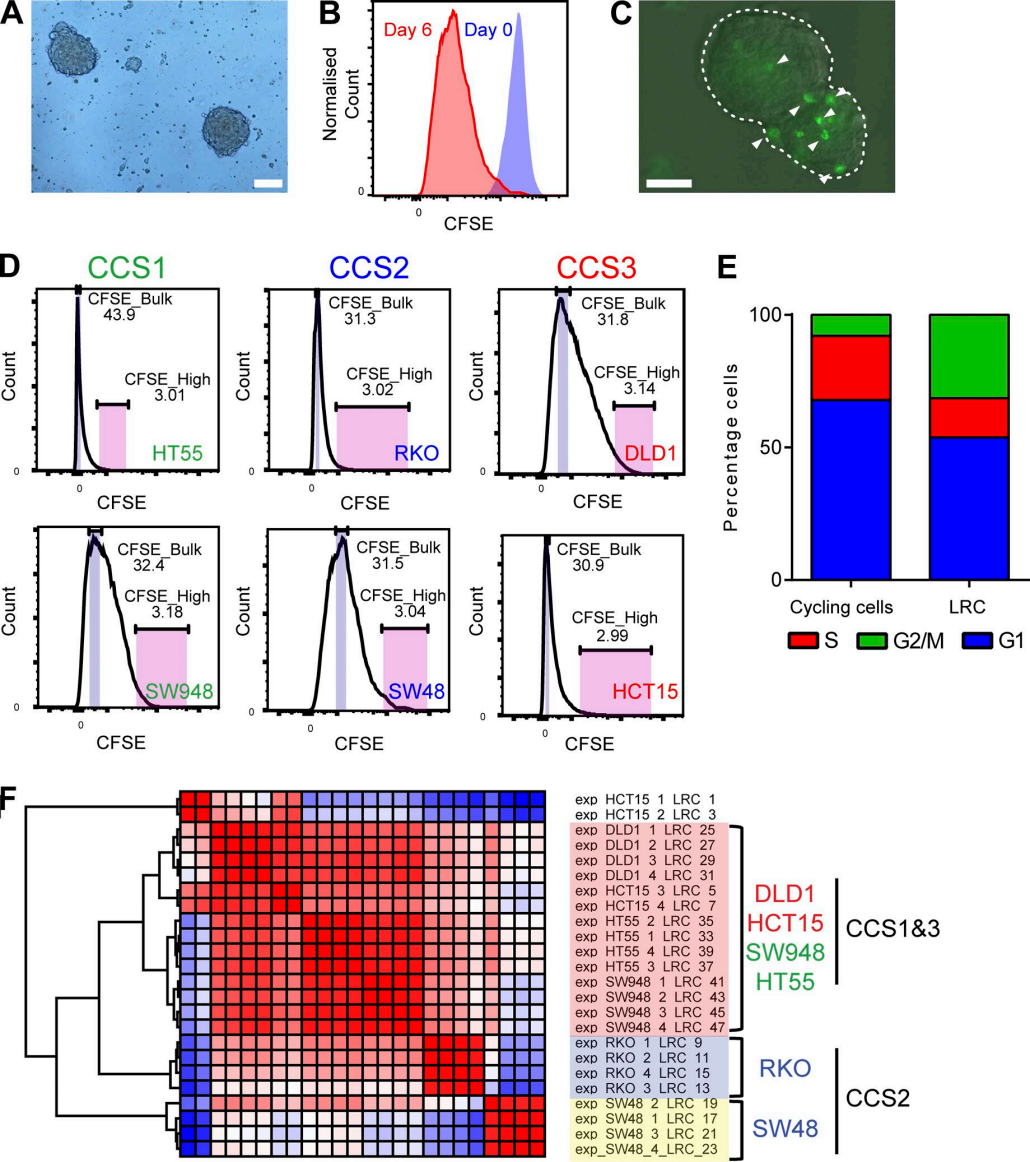
**Dormant tumor cells may be isolated from spheroid culture using dye staining with subsequent chase. (A)** Bright field image of HCT15 spheroids 5 d after seeding. **(B)** FACS histogram from SW48 spheroid cells after CFSE labeling (blue) and 6 d later (red). **(C)** Image of HCT15 spheroid 6 d after labeling with CFSE. Single CFSE^High^ (LRC) cells indicated. Bars, 100 µm (A and C). **(D)** FACS histograms of CFSE levels in 6 CRC cell lines 6 d after CFSE labeling. **(E)** Stacked bar chart showing the cell cycle status of SW948 spheroid cycling and LRCs. **(F)** Hierarchical clustering of RNAseq data for LRCs from each cell line.

Dormant cells have previously been identified in CRC cell lines and xenografts using dye staining and subsequent dilution assays and are classified as label-retaining cells (LRCs) if the dye is retained with time (Moore et al., 2012). To isolate and characterize CRC LRCs, we applied this approach using CFSE in 3D nonadherent serum-free spheroid culture (Fig. 1, A–C), together with FACS and transcriptomic profiling. Six human CRC cell lines representing the three main CRC subtypes (CCS1–3) were analyzed to identify potential subgroup specific patterns (Fig. 1 D; De Sousa E Melo et al., 2013; Linnekamp et al., 2018). The colon cancer subtype (CCS) classification overlaps with the recent CRC consensus classification where CCS1 = CMS2/3, CCS2 = CMS1, and CCS3 = CMS4. All lines displayed high initial CFSE staining and then dilution with time (Fig. 1 B). LRCs were visible in cultured spheroids up to 2 wk after initial seeding, compatible with a previous study for an appropriate time point to isolate tumor LRCs (Fig. 1 C; Pece et al., 2010). Cell cycle analysis of CFSE-labeled SW948 spheroids 1 wk after labeling showed a higher number of LRCs in G2/M and less in S phase, compared with cycling cells compatible with previous in vivo studies in CRC (Fig. 1 E; Moore et al., 2012). Mathematical modeling (see Materials and methods) of SW48 CFSE dilution kinetics predicted the presence of two populations, one rapidly cycling and a smaller (<2%) slowly cycling population (CFSE^High^; Fig. S1, A and B).

**Figure 2.**
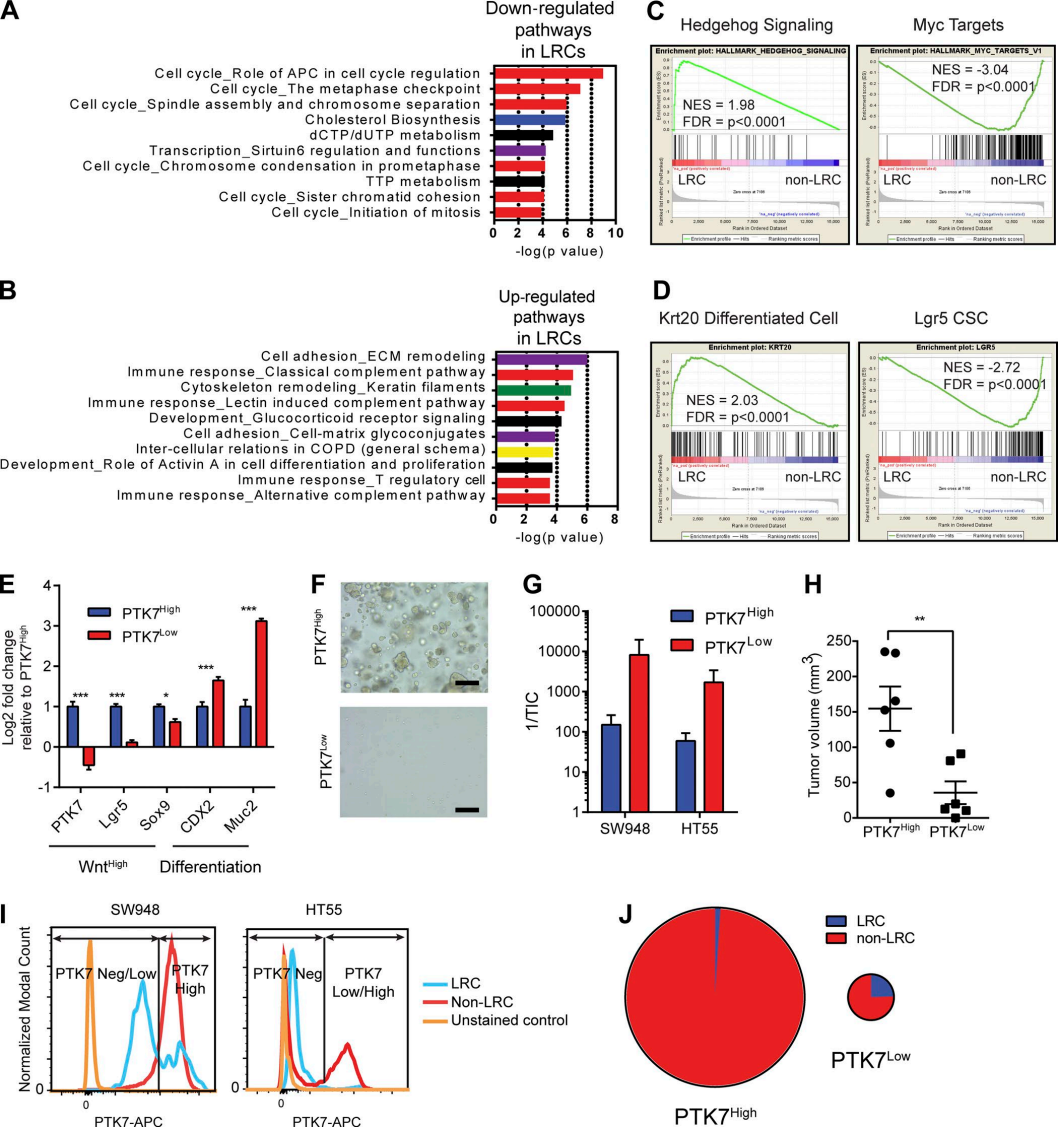
**Dormant CRC cells are a differentiated cell population. (A)** GO pathway analysis of genes down-regulated in LRCs. **(B)** GO pathway analysis of genes up-regulated in LRCs. **(C)** GSEA charts of significantly enriched gene sets in LRCs and non-LRCs. **(D)** GSEA showing enrichment of the Krt20 differentiated gene set in LRCs and the Lgr5 CSC gene set in non-LRCs. **(E)** RT-PCR histogram showing enrichment of Wnt targets in PTK7^High^ and differentiation markers in PTK7^Low^. *n* = 6; mean ± SEM. ***, P < 0.001; *, P < 0.05 by two-way ANOVA. **(F)** Bright field images of PTK7^High^ and PTK7^Low^ SW948 spheroid cells 5 d after seeding in nonadherent culture. Bars, 100 µm. **(G)** Histogram of the tumor-initiating cell frequency (TIC) from FACS sorted SW948 and HT55 spheroids. Mean ± SEM. **(H)** Column scatter plot of xenograft sizes derived from PTK7^High^ and PTK7^Low^ SW948 cells. Mean ± SEM; **, P < 0.01 by unpaired *t* test. **(I)** FACS histogram of PTK7 levels in LRCs and non-LRCs derived from CFSE-labeled SW948 and HT55 spheroids. **(J)** Pie charts of the relative proportions of LRCs and non-LRCs within PTK7^High^ and PTK7^Low^ populations from SW948 spheroids. Size of each chart is proportional to relative numbers of cells present.

To identify targetable pathways and generate a molecular signature from LRCs in spheroid culture, we performed RNA sequencing (RNAseq) on day 6 FACS-sorted LRC (CFSE^High^) and non-LRC (CFSE^Bulk^) populations (Fig. 1 D). Hierarchical clustering showed that cell lines clustered primarily according to subtype and secondarily to CFSE retention (Fig. S1 C). CCS1^+^3-LRCs were most similar in expression profile while CCS2-LRCs clustered separately (Fig. 1 F). As predicted by their LRC status, gene ontology (GO) pathways associated with down-regulated genes were dominated by cell cycle components (Fig. 2 A). GO pathway analysis of overexpressed genes in LRCs across subtypes demonstrated strong representation from pathways involved in the immune response (complement pathway), cell adhesion, and cytoskeletal remodeling (Fig. 2 B). Gene set enrichment analysis (GSEA) showed the most significant hallmark gene set enriched in LRCs was implicated in hedgehog (Hh) signaling, whereas the most underrepresented gene set was associated with targets of Myc (Fig. 2 C). Matrix Metalloproteinases and Keratins were abundantly expressed in LRCs, suggesting that LRCs may represent a differentiated cell population. To test for similarities with differentiated CRC cell signatures, GSEA was performed on the microarray expression dataset generated from the recent Shimokawa study (Shimokawa et al., 2017). The top 200 genes expressed in Lgr5^+^ CSCs and Krt20^+^-differentiated cells were compared with the LRC transcriptome (Data S1 and Fig. 2 D). There was a highly significant enrichment of the differentiated cell signature with the LRC transcriptome. Conversely, there was an enrichment of the Lgr5 CSC signature with non-LRCs (Fig. 2 D). We also noted, using our RNAseq data, that mRNA levels of Wnt target genes were elevated in CCS1-LRCs when compared with CCS2- and 3-LRCs, compatible with the overall higher Wnt activity reported in this subtype (Fig. S1 D). However, LRCs from all subtypes (including CCS1) had lower levels of Wnt target gene expression than non-LRCs.

To validate the Krt20/Lgr5 GSEA findings (Fig. 2 D), FACS was used using a CSC-specific marker. From the Sato microarray data for Lgr5^+^ CSCs, we identified a potential antibody based marker founded on the newly described human colon stem cell marker PTK7 (Data S1; Jung et al., 2015; Shimokawa et al., 2017). In the normal colon, PTK7^High^ marks the Wnt^High^ Lgr5^+^ stem cell compartment and PTK7^Neg/Low^, a nonclonogenic differentiated population. To ascertain whether PTK7 marks similar populations in human CRCs, FACS was performed for PTK7^High^ and PTK7^Low^ populations from SW948 spheroids, and then RT-PCR was performed for Wnt target genes (Lgr5 and EphB2) and differentiation markers (CDX2 and Muc2). RT-PCR confirmed PTK7^High^ and PTK7^Low^ mark a stem-like Wnt^High^ population and a differentiated population, respectively (Fig. 2 E). It was noted that when PTK7^High^ cells were grown in spheroid culture, they had far higher spheroid-forming efficiency than PTK7^Low^ cells (Fig. 2 F). To quantify these differences, extreme limiting dilution analysis (LDA) was performed using PTK7^Low^ and PTK7^High^ cells from SW948 and HT55 spheroids to identify spheroid forming efficiencies (Fig. 2 G). LDA demonstrated that PTK7^High^ cells from both cell lines had a higher tumor-/spheroid-initiating cell (TIC) frequency than PTK7^Low^ cells. Next, we sought to establish whether PTK7^High^ cells were more proliferative in vivo than PTK7^Low^ cells compatible with their Wnt^High^ CSC phenotype. PTK7^High^ and PTK7^Low^ cells were sorted from SW948 spheroids, and 50,000 cells (to ensure adequate tumor formation from both populations) were engrafted bilaterally into NSG mice. Mice were left for 5 wk to establish subsequent tumor size. Tumors grew in all mice engrafted with PTK7^High^ cells and all but one of the PTK7^Low^-engrafted animals, compatible with the previously calculated TIC frequencies. Tumors derived from PTK7^High^ cells were found on average to be larger than those from PTK7^Low^ cells (*n* = 6; Fig. 2 H and Fig. S1 E)

Having validated PTK7 expression as a novel CSC marker and a read-out of Wnt activity, FACS quantification of PTK7 levels was performed from single cells derived from CFSE-labeled CCS1-SW948 and HT55 6-d spheroids, which confirmed LRCs to generally be marked by PTK7^Low^ and non-LRCs PTK7^High^ (Fig. 2 I). Validating the GSEA analyses in SW948 cells, it was found that 64% ± 2% of LRCs were found to be PTK7^Neg/Low^, whereas 82% ± 2% of non-LRCs were PTK7^High^; that is, the majority of LRCs have lower levels of PTK7 than non-LRCs (Fig. 2 I). Further, of the PTK7^Neg/Low^ population, 24.5% ± 3% were LRCs, implying that LRCs are a subpopulation of differentiated tumor cells, yet the majority (98.8% ± 0.1%) of PTK7^High^ cells are cycling cells (Fig. 2 J).

**Figure 3.**
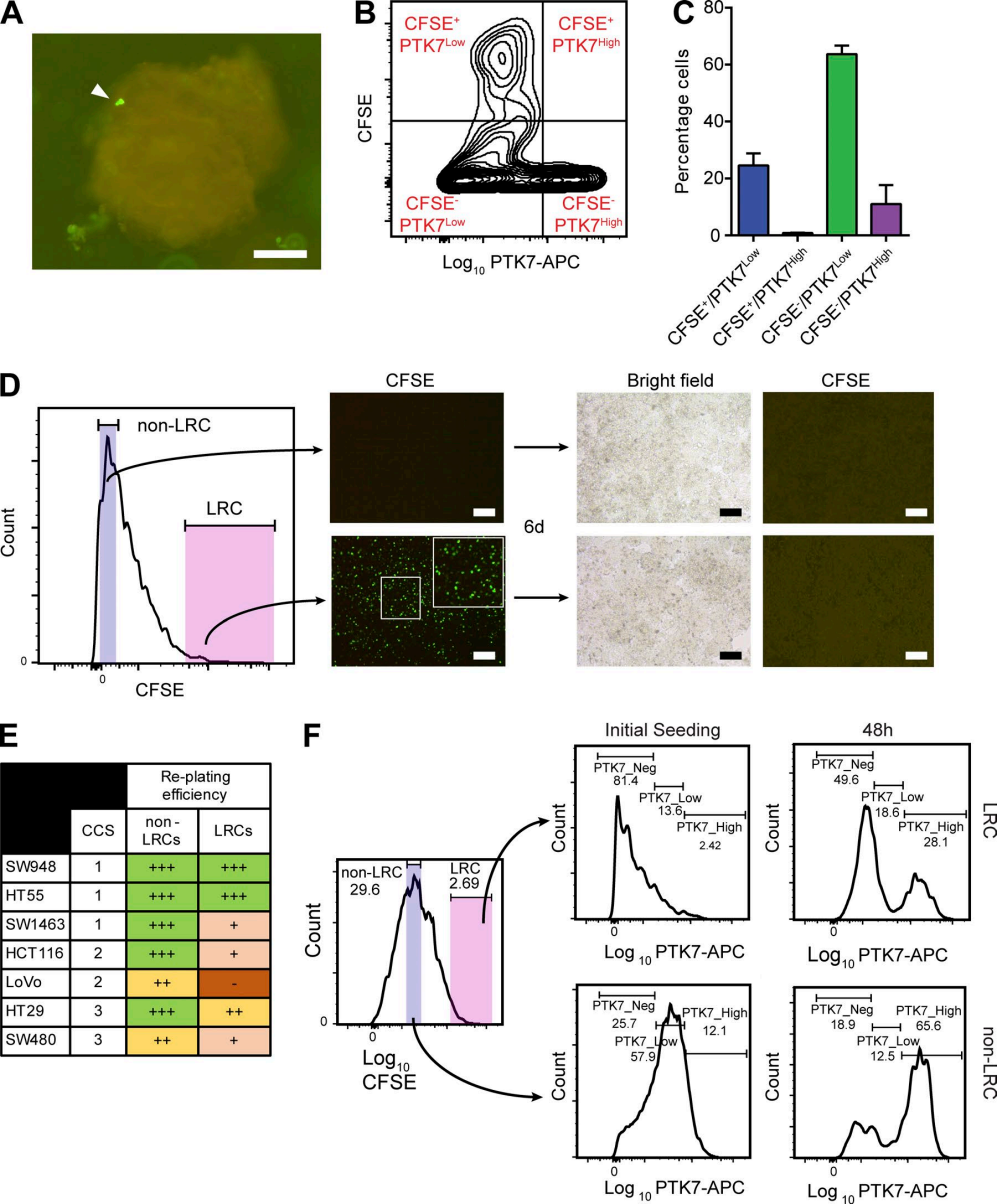
**CCS1-LRCs are differentiated yet can revert to a CSC-like state. (A)** Fluorescence micrograph of CFSE-labeled PDO (COLO05) 8 d after labeling with CFSE. LRC marked with arrow. **(B)** Representative FACS density plot of PTK7 levels in CFSE labeled PDOs. **(C)** Histogram of B showing the majority of LRCs (CFSE^+^) are differentiated (PTK7^Low^). **(D)** FACS histogram showing the sorting strategy for reseeding of SW948 CFSE-labeled spheroid-derived LRCs and non-LRCs. Accompanying fluorescent microscopy images from seeded wells. Bars, 100 µm (A and D). **(E)** Replating efficiencies of LRCs and non-LRCs across CCSs. **(F)** FACS quantification of changes in differentiation status (PTK7 levels) in SW948 spheroid LRCs and cycling cells upon replating in adherent culture.

To ascertain whether LRCs derived from primary CRCs were similar to those from CRC cell lines, we made use of patient-derived organoid (PDO) culture. Three CRC PDO lines were generated from primary tissue and cultured as previously reported (van de Wetering et al., 2015). Targeted sequencing showed that all three lines were derived from the common CCS1 subtype (Data S1 and Materials and methods). PDOs were passaged, and cells were labeled with CFSE as described earlier. After 8 d in culture, organoids were disaggregated and cells underwent flow cytometric analysis of CFSE and PTK7 levels (Fig. 3, A–C). In line with the results from the cell lines, the vast majority (97% ± 0.6%) of PDO-LRCs from all lines retained a PTK7^Neg/Low^ differentiated cell phenotype (Fig. 3, B and C).

It has previously been established that CRC LRCs form xenografts in vivo, although the mechanism behind this apparently paradoxical behavior is not known (Moore et al., 2012). We sought to understand whether LRCs in vitro retain the same ability to proliferate and, if so, to use these assays to describe the type of phenotypical change seen. CFSE-labeled SW948 cells were grown as spheroids, and then FACS was performed for LRCs (CFSE^High^) and non-LRCs (CFSE^Bulk^). Equal cell numbers of each sorted population were then seeded in permissive 2D culture to quantify latent proliferative capacity (Fig. 3 D). Both LRCs and non-LRCs retained the ability to proliferate after sorting, despite differences in initial cell cycle status (Fig. 1 E). Both populations proliferated within 24 h of seeding, and full confluency was achieved in 6 d for CFSE^Bulk^ and 7 d for CFSE^High^ populations, implying an acquisition of near-similar cycling speed in LRCs (Fig. 3 D). There were only very rare, scattered remaining LRCs in the CFSE^High^-sorted populations by the time confluency was achieved. Next, both populations were relabeled with CFSE and reseeded in 3D spheroid culture (Fig. S1 F). Both populations retained the capacity to generate spheroids with new LRCs and non-LRCs derived from both populations (Fig. S1 F). To test whether LRCs from other CCSs also retained the capacity for proliferation, similar experiments were performed with spheroid-derived LRCs and non-LRCs. Although most LRCs retained an ability to proliferate, only LRCs from CCS1 SW948 and HT55s proliferated as efficiently as non-LRCs (Fig. 3 E). To identify whether LRCs from the CCS1 subtype underwent dedifferentiation to a stem cell state on replating, we measured PTK7 levels in LRCs and non-LRCs at the time of and 48 h after replating (Fig. 3 F). Both LRCs and non-LRCs acquired higher levels of PTK7 after replating, confirming dedifferentiation to a Wnt^High^ stem–like state. In summary, the LRC cell cycle status in CCS1 tumors is highly plastic, and the population retains the ability to dedifferentiate and proliferate, analogous to the behavior previously described for LRCs in the normal epithelium and also Krt20-differentiated tumor cells’ behavior in vitro (Buczacki et al., 2013; Shimokawa et al., 2017).

Cumulatively, these data show that tumor LRCs are a differentiated cell population that express Hh components, but additionally, in CCS1 tumors, they are also found to express higher levels of Wnt target genes than LRCs from CCS2&3. To further identify and test pathways potentially involved in regulating this dormancy, a screen of compounds including Hh and Wnt manipulators was undertaken.

### Itraconazole releases dormancy by inducing tumor-wide proliferation then arrest

**Figure 4.**
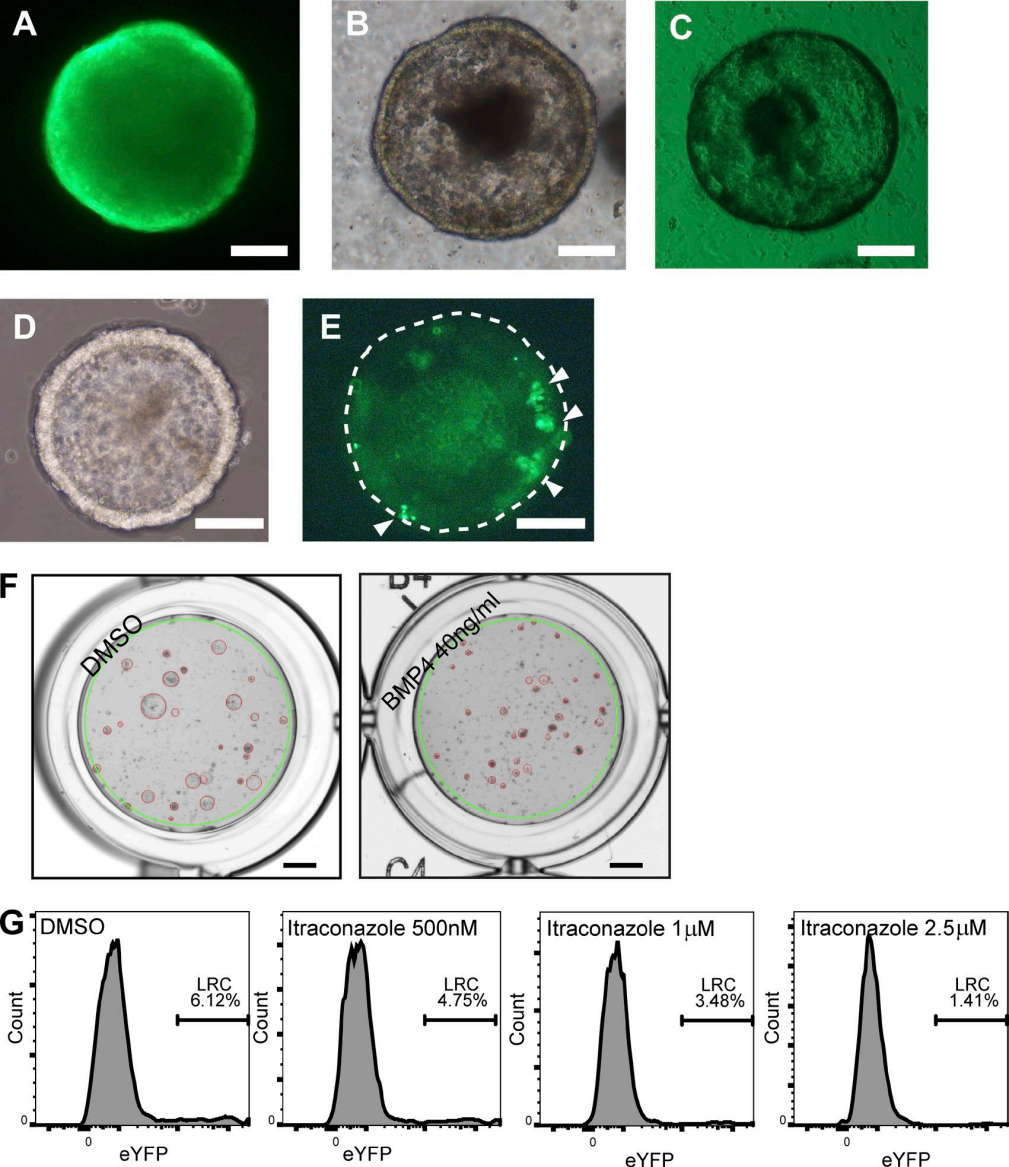
**A 3D tumor organoid drug screen using label-retaining Ah-H2B-YFP_Apc1322t mice identifies modulators of spheroid growth and cellular dormancy. (A–E)** Fluorescence and bright field microscopy images of βNF induced YFP-1322 organoids 24 h after βNF treatment (A and B), uninduced control (C), and 5 d after βNF treatment (D and E). White arrowheads show retained CFSE in LRCs, and the dashed line demonstrates the outer border of the organoid (E). **(F)** Example images of control treated organoids (left) and positive control (right) BMP4 treated and collapsed organoids. The green line indicates the uniformly applied well mask and the red circles indicate the organoids identified by a customized CHARM setting. Bars: (A–E) 100 µm; (F) 1 mm. **(G)** Example flow cytometry histogram plots of cellular YFP intensity (Single/PI-) from disaggregated organoids 5 d after βNF treatment and 4 d after treatment with carrier control or itraconazole at 500 nM, 1 µM, or 2.5 µM.

**Figure 5.**
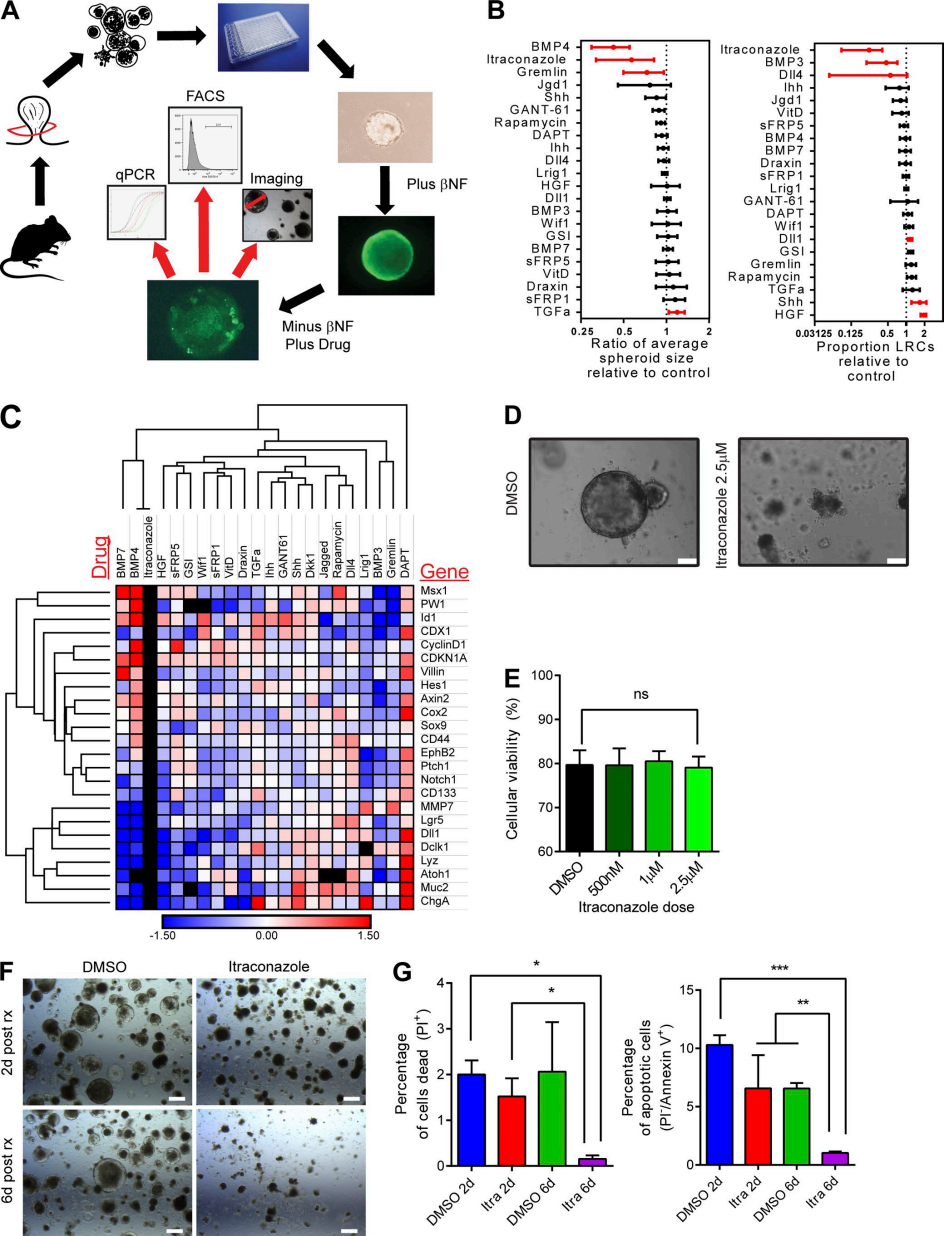
**A mouse tumor organoid drug screen identifies itraconazole as a modulator of quiescence and adenoma organoid development. (A)** Schematic of the drug screen protocol using YFP-1322 mice. **(B)** Column graphs of the effect of candidate drugs on organoid size and proportion of LRCs. Mean ± SD. Significant (P < 0.05 by independent *t* tests on each drug treatment compared with control) changes shown in red. **(C)** Hierarchical clustering of RT-PCR changes in (cancer) stem, differentiation, and pathway associated genes. Red, upregulated. Blue, down-regulated. Black, not detected. **(D)** Bright field images of YFP-1322 tumor organoids 10 d after single cell plating and 4 d after treatment with DMSO or itraconazole. Bars, 100 µm. **(E)** Percentage viability of organoid cells as determined by PI incorporation after itraconazole treatment. *n* = 3; mean ± SD; ns, not significant by one-way ANOVA). **(F)** Bright field images of Apc-deficient mouse spheroids treated with itraconazole (2.5 µM) or DMSO at early and late time points after treatment. Bars, 100 µm. **(G)** Histograms of changes in viability (PI) and apoptosis (Annexin V) in mouse spheroids after treatment with itraconazole at early and late time points. *n* = 3; mean ± SEM; *, P < 0.05; **, P < 0.01; ***, P < 0.001 by one-way ANOVA.

To focus our analysis to a biologically reproducible tumor type with a single genetic etiology and identify candidate compounds and pathways that could facilitate cell cycle reentry in LRCs, a mouse 3D organoid drug screen was used (Fig. 4 and Fig. 5 A). Mouse tumor organoids derived from *Apc^1322t^* mice (that, like the most common CCS1 subtype, show high Wnt signaling) were used in combination with small molecules and recombinant proteins to analyze the effects of manipulation of the Wnt, Hh, BMP, Notch, epidermal growth factor (EGF)/ErbB, and mTOR pathways on tumor development.

*Apc^1322t^* mice were crossed with *Ah-H2B-YFP* transgenic mice (Buczacki et al., 2013). Spontaneous intestinal tumors from double transgenic animals (YFP-1322) were excised, and cells were isolated to generate primary organoid cultures that allowed a YFP-pulse, with in vitro βNF induction and subsequent chase to identify LRCs (Fig. 4, A–E). Adenoma organoids were cultured as previously described by Sato et al. (2011a). Uninduced organoids demonstrated no YFP expression (Fig. 4 C). Scattered LRCs were identified in induced organoid cultures up to 8 d after removal of βNF (Fig. 4, D and E).

Next, organoids received candidate treatments likely to affect cell cycle progression (see Materials and methods and Data S1). Organoids were then analyzed for differentiation effects using RT-PCR, the proportion of remaining LRCs using flow cytometry, and organoid growth using an automated imaging system (Fig. 4, F and G). Predictably, as a result of the presence of a downstream Apc mutation, Wnt ligand antagonism with four separate compounds (Wif1, sFRP1, sFRP5, and Draxin) failed to generate any culture phenotype (Fig. 5 B). RT-PCR quantification showed that several compounds (BMP7, BMP3, HGF, and DAPT) induced profound changes in secretory differentiation (Fig. 5 C). However, despite large molecular differentiation effects being acquired with pathway manipulation (Fig. 5 C), overall organoid growth was generally unaffected across conditions, implying significant functional cellular redundancy and plasticity (Fig. 5 B). The most dramatic changes seen were with attempted Hh manipulation using itraconazole (a previously described smoothened antagonist) that generated almost complete loss of LRCs and marked organoid growth collapse (Fig. 5, B and D), but with no change to overall cellular viability (Fig. 5 E). To test whether cells were initially entering apoptosis and then surviving cells were arresting, we performed apoptosis (Annexin V) and viability (PI) staining at early (2 d) and late time points (6 d) in organoids treated with itraconazole (Fig. 5, F and G). As described earlier, early time points demonstrated no change in overall viability; however, at 6 d after treatment, after the spheroid collapse had occurred, there were significantly fewer dead and apoptotic cells in itraconazole-treated samples, suggesting global cell cycle arrest had developed. From the drug screen, as a result of the limited numbers of cells present in wells treated with high dose (2.5 µM) itraconazole, no reliable RT-PCR measurements were available to suggest the molecular mode of action of the drug (Fig. 5 C). However, importantly, Gant-61, another well-validated canonical Hh antagonist acting downstream of smoothened, failed to reproduce the same phenotype, suggesting an alternative, non–Hh-driven explanation for the effect seen (Fig. 5 B).

**Figure 6.**
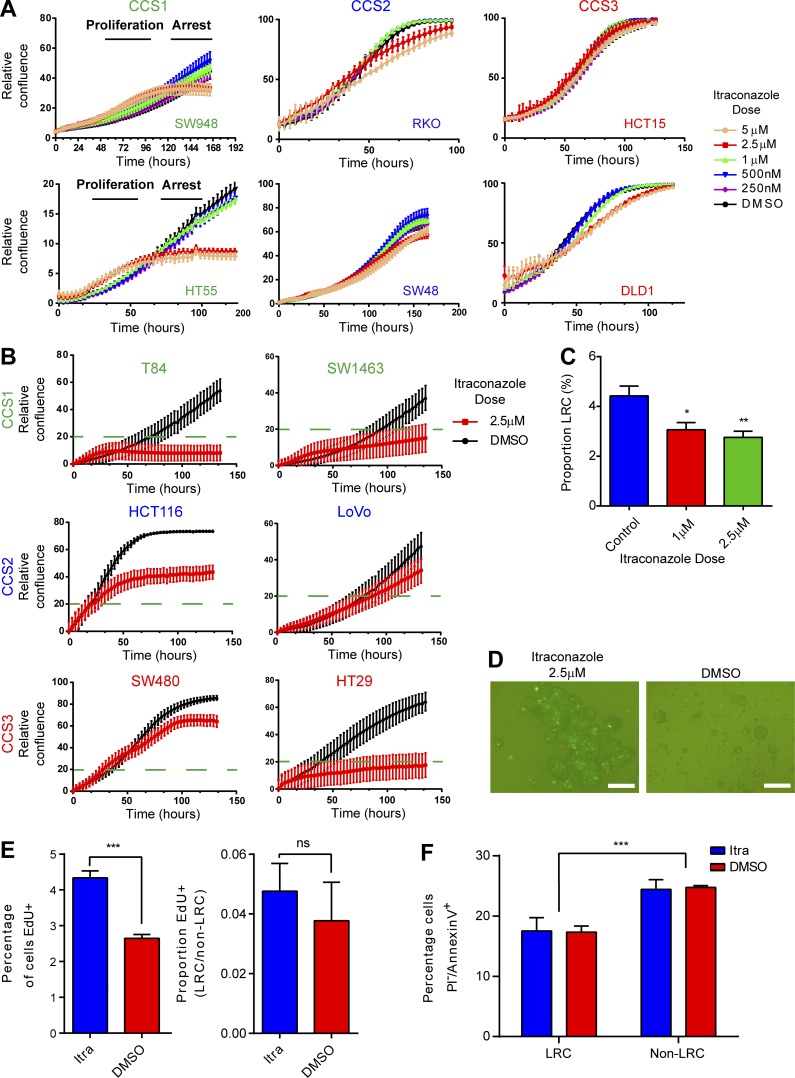
**Itraconazole treatment of CRC cell lines demonstrates treatment is effective in CCS1 cell lines and eliminates dormant cells. (A)** Live cell confluence graphs of six CRC cell lines grown in 2D showing a dose-dependent effect in response to itraconazole in SW948 and HT55 lines. **(B)** Validation live cell confluence graphs for six CRC further cell lines. Full sensitivity (confluence <20%) was seen in T84 and SW1463 cells (CCS1) and HT29 cells (CCS3). **(C)** FACS measurements of the proportions of LRCs in CFSE labeled SW948 spheroids 4 d after treatment with itraconazole or DMSO. *n* = 3; mean ± SEM; *, P < 0.05 by one-way ANOVA. **(D)** Fluorescent images of SW948 spheroids grown in nonadherent culture after CFSE labeling for 4 d and then treated with itraconazole or DMSO for 48 h. Bars, 100 µm. **(E)** Histograms quantifying EdU incorporation in SW948 spheroids treated with itraconazole or DMSO during the proliferative burst. *n* = 3; mean ± SEM; ***, P < 0.001 by unpaired *t* test. **(F)** Histogram displaying levels of apoptosis (PI^−^/AnnexinV^+^) in HT55 CFSE labeled spheroids 10 d after labeling and 48 h after treatment with itraconazole or DMSO. *n* = 3; mean ± SEM; ***, P < 0.001 by two-way ANOVA; ns, not significant.

To validate the itraconazole-induced phenotype, the six human CRC cell lines used previously were treated with itraconazole, and responsiveness was quantified using continuous 2D live cell confluence measurements. Two of the six lines (HT55 and SW948 [CCS1]) demonstrated responsiveness (Fig. 6 A). These results were validated on six further cell lines where full sensitivity (confluence <20%) was seen again in both CCS1 cell lines (T84 and SW1463) and only partial or nonresponse in CCS2 (HCT116 and LoVo) and CCS3 (SW480; Fig. 6 B). HT29 cells (CCS3) also demonstrated a strong response to itraconazole treatment. Characteristically, an initial proliferative phase was seen, followed by apparent cell cycle arrest, potentially explaining the initial loss/mobilization of LRCs seen in mouse spheroids.

To test whether LRCs were also lost in human CRCs, SW948 cells were labeled with CFSE and grown as spheroid cultures; 4 d later, they were treated with itraconazole or control. 4 d further after treatment, spheroids were disaggregated, and FACS analysis was performed for the proportion of CFSE^High^ cells remaining (LRCs; Fig. 6, C and D). As predicted by the live cell confluence experiments, high dose itraconazole initially reduced the proportion of LRCs. To ascertain whether itraconazole forces cell cycle progression differentially between LRCs and non-LRCs, EdU incorporation was quantified in CFSE-labeled and itraconazole or DMSO-treated SW948 spheroids (Fig. 6 E). After labeling with CFSE, cells were grown to generate spheroids with both LRCs and non-LRCs as described earlier. 10 d after seeding, spheroids were treated with itraconazole or DMSO. 36 h later (to capture the proliferative burst) spheroids were provided with 1 h of EdU exposure. Spheroids then underwent flow cytometric quantification of EdU incorporation. Analogous to the proliferative burst seen in 2D culture, larger numbers of cells in spheroid culture incorporated EdU shortly after treatment with itraconazole. However, this effect of itraconazole is equally distributed between LRCs and non-LRCs (Fig. 6 E). To ascertain whether LRCs were dying as a result of itraconazole treatment, Annexin V levels were quantified in HT55 tumor spheroids 10 d after CFSE labeling and 48 h after treatment with itraconazole. Levels of apoptosis (PI^−^/Annexin V^+^) were higher in non-LRCs than LRCs compatible with their proliferative nature (Fig. 6 F). However, itraconazole treatment did not alter the number or distribution of apoptotic cells between LRCs and non-LRCs, further confirming proliferation then arrest rather than cell death as the phenotype associated with itraconazole treatment. Finally, pulse-labeling cells grown in 2D, with EdU in combination with propidium iodide (PI)–staining at both 2 and 7 d (see Materials and methods) after itraconazole treatment confirmed that cells initially stimulated to enter the cell cycle (EdU^+^) by itraconazole treatment subsequently growth arrest in G1 (Fig. S2, A and B).

Overall, these data show that itraconazole treatment alters cell cycle progression in both dormant and nondormant cells by initially causing proliferation and subsequently G1 cell cycle arrest. Not all tumors responded, however, and treatment efficacy appeared most pronounced in the CCS1 subtype that is characterized by high Wnt levels. We therefore sought to ascertain whether Wnt signaling, Lgr5 status, or alternative pathways were effected by itraconazole treatment.

### Itraconazole treatment inhibits Wnt signaling and induces global senescence in responsive CCS1 cell lines

**Figure 7.**
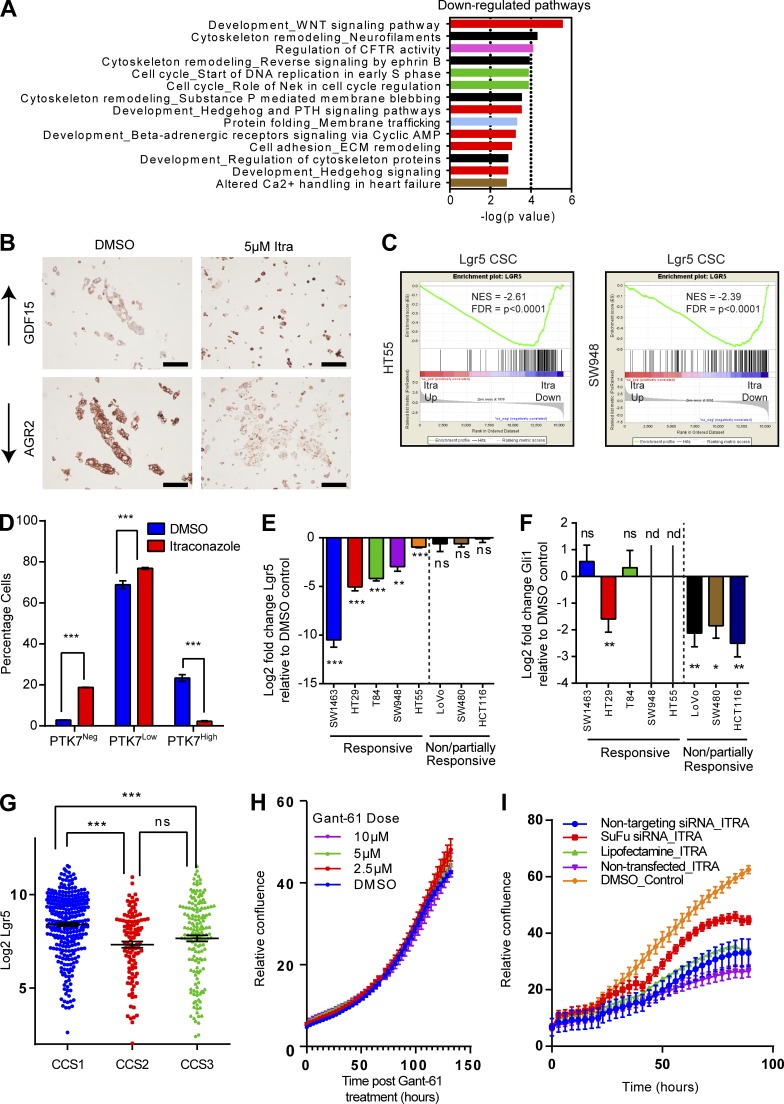
**Itraconazole treatment inhibits Wnt signaling. (A)** GO pathway analysis of differentially expressed genes in HT55 and SW948 cells after itraconazole treatment. **(B)** Immunohistochemical validations of RNAseq data of itraconazole treatment in SW948 cells. Bars, 100 µm. **(C)** GSEA showing loss of the Lgr5 CSC signature in response to itraconazole treatment in HT55 and SW948 cells. **(D)** Histogram of changes in anti-PTK7 staining levels from SW948 spheroids treated with itraconazole (2.5 µM) or DMSO control. *n* = 3; mean ± SEM. ***, P < 0.001 by two-way ANOVA. **(E)** RT-PCR quantification of Lgr5 levels observed with itraconazole treatment in eight CRC lines. *n* = 4; mean ± SEM. **, P < 0.01; ***, P < 0.001; ns, not significant by one-way ANOVA. **(F)** RT-PCR quantification of Gli1 levels observed with itraconazole treatment in eight CRC lines. *n* = 4; mean ± SEM. *, P < 0.05; **, P < 0.001; ns, not significant by one-way ANOVA; nd, transcript not detected. **(G)** Lgr5 expression levels across CRC subtypes from Marisa et al. (2013) dataset. ***, P < 0.001 by one-way ANOVA; ns, not significant. **(H)** Live cell confluence graph of the response of SW948 cells to Gant-61 treatment. **(I)** Live cell confluence measurements of the response of SW948 cells to itraconazole in the presence of siSuFu.

Itraconazole, a commonly prescribed antifungal, has been shown to antagonize canonical Hh signaling in medulloblastoma and basal cell carcinoma. Itraconazole inhibits smoothened, a key mediator of the Hh pathway, by binding at an unknown location, but distinct from that of other classical smoothened inhibitors (Kim et al., 2010). Our cell line responsiveness neither correlated with Hh component mutational status, copy number, nor genes previously associated with itraconazole resistance (unpublished data; Barretina et al., 2012). Therefore, to better understand the molecular effects of itraconazole, RNAseq analysis was performed on itraconazole-treated HT55 and SW948 cell lines 6 d after treatment. GO pathway analysis of gene expression changes showed significant down-regulation in both the Wnt and Hh signaling pathways, as well as cell cycle–associated pathways, but no evidence of autophagy, mTOR, or MAPK changes also previously associated with itraconazole treatment (Fig. 7 A; Liu et al., 2014; Hara et al., 2016). Selected highly altered genes were validated using immunohistochemistry including AGR2 (a known correlate of Wnt activity; Fig. 7 B; Valladares-Ayerbes et al., 2012). Strikingly, using GSEA, there was a highly significant loss of the Wnt^High^ Lgr5^+^ CSC signature upon treatment with itraconazole in both cell lines (Fig. 7 C; Shimokawa et al., 2017). To validate these GSEA findings, SW948 cells were grown as spheroids for 4 d and then treated with itraconazole for 3 d. Spheroids were then disaggregated, and FACS was performed to quantify the proportion of Wnt^High^ CSCs using anti-PTK7 staining (Fig. 7 D). Itraconazole treatment was found to diminish PTK7 levels in all cells, confirming loss of the Wnt^High^ CSC phenotype.

**Figure 8.**
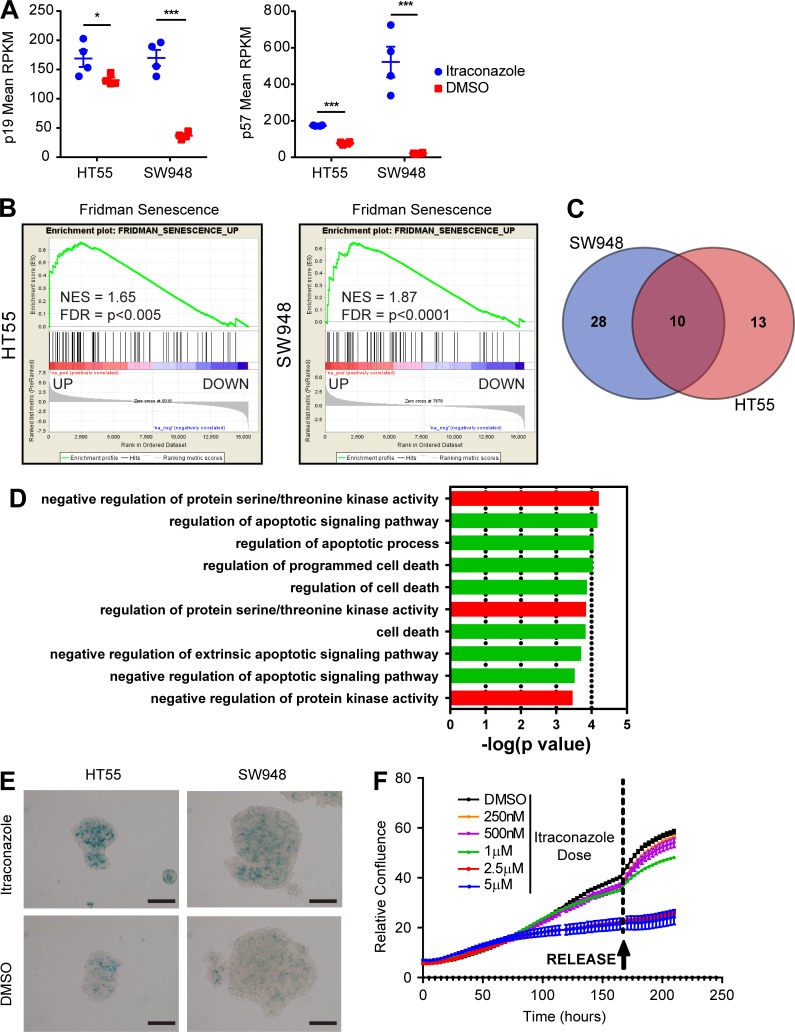
**Itraconazole treatment induces cellular senescence in responsive CCS1 cell lines. (A)** Dot graphs showing normalized levels (reads per kilobase million [RPKM]) of p19 (CDKN2D) and p57 (CDKN1C) derived from RNAseq data in HT55 and SW948 cells treated with DMSO or itraconazole (mean ± SEM; ***, P < 0.001; *, P < 0.05 by independent *t* tests on each cell line. **(B)** GSEA plots for the association between genes regulated by itraconazole and those defining senescence in HT55 and SW948 cells. **(C)** Venn diagram showing the overlap of known senescence-associated genes (Fridman) differentially expressed in response to itraconazole treatment in HT55 and SW948 cells. **(D)** GO Processes associated with the core itraconazole senescence gene set. **(E)** Bright field images of HT55 and SW948 cells treated with itraconazole (2.5 µM) or DMSO and stained with SA-β-GAL. Bars, 100 µm. **(F)** Live cell confluence measurements of HT55 cells treated with itraconazole for 180 h and then “released” from treatment by media exchange to standard growth conditions.

*CDKN1C* (*p57*) and *CDKN2D* (*p19*), previously shown to be associated with senescent cells, were found elevated in both cell lines in response to itraconazole (Fig. 8 A; Matsuoka et al., 1995; Kang et al., 2003; Giovannini et al., 2012). To investigate if senescence is induced after itraconazole treatment, the RNAseq data were compared with the senescence-defining dataset of Fridman (Fridman and Tainsky, 2008). There was a large overlap of the entire Fridman dataset to genes similarly expressed in response to itraconazole treatment; >50% (43/77) of the identified Fridman genes were found differentially expressed in SW948 cells and >30% (24/77) in HT55 cells. There was a statistically significant correlation of up-regulated Fridman genes and up-regulated expression in both cell lines (P < 0.0001), and this finding was further confirmed using GSEA (Fig. 8 B). 10 genes were identified in both cell lines to be differentially expressed in response to itraconazole and associated with senescence (Fig. 8 C and Data S1). GO Process analysis of this core itraconazole senescence-associated gene set showed strong representation of processes involved in the negative regulation of cell death and apoptosis compatible with the senescence phenotype seen (Fig. 8 D).

To experimentally test whether itraconazole-arrested cells had entered senescence, senescence-associated β-galactosidase (SA-β-GAL) staining was performed on HT55 and SW948 cells in 2D culture 6 d after itraconazole treatment. High levels of SA-β-GAL staining were seen in treated cells, confirming the induction of senescence (Fig. 8 E). Importantly, proliferative arrest remained after removal of itraconazole, further confirming the senescent phenotype (Fig. 8 F).

**Figure 9.**
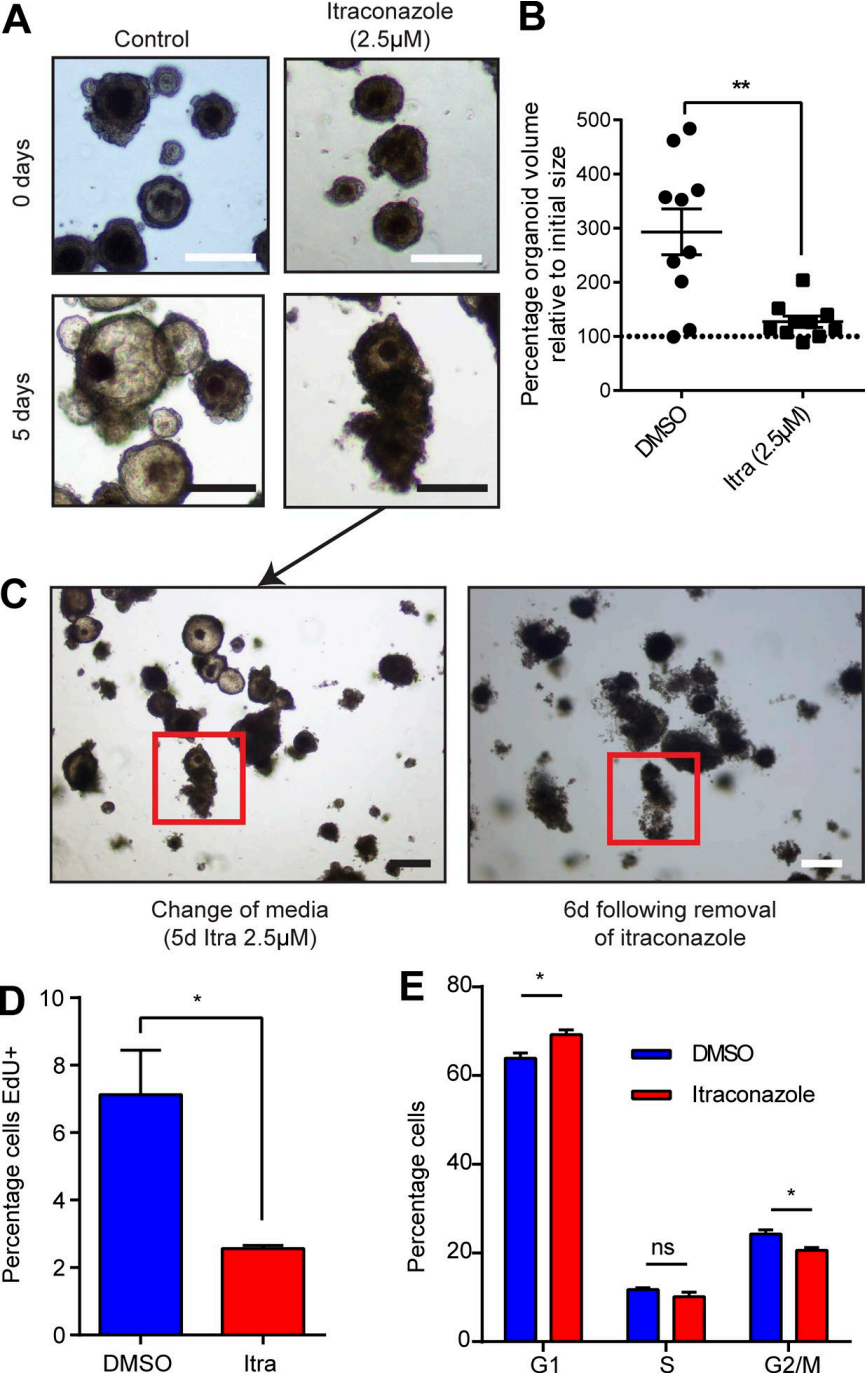
**Itraconazole treatment induces phenotypically similar changes in primary PDOs to that seen in cell lines. (A)** Representative bright field images of changes in PDO morphology in response to itraconazole treatment. Bars, 100 µm. **(B)** Dot plot quantification of the change in PDO volume in response to itraconazole treatment or DMSO. *n* = 3; mean ± SEM; **, P < 0.01 by two-tailed Mann-Whitney test. **(C)** Bright field–matched images demonstrating ongoing response to itraconazole treatment after removal of treatment. Bars, 100 µm. **(D)** Histogram of EdU incorporation in PDOs 5 d after itraconazole treatment or control. *n* = 3; mean ± SEM; *, P < 0.05 by unpaired *t* test. **(E)** Histogram showing cell cycle distribution of PDO cells after 5 d of itraconazole treatment or control. *n* = 3; mean ± SEM; *, P < 0.05; ns = not significant by independent *t* tests on each cell cycle stage).

Next, PDOs were assessed for similar responses to itraconazole treatment. PDOs were established and treated with itraconazole or control. All three PDO lines responded phenotypically in a similar manner to mouse organoids, with organoid growth collapse occurring approximately 4 d after treatment (Fig. 9, A and B). To ascertain whether treatment was reversible in PDOs, organoids were treated with itraconazole for 5 d, and then media were replaced without the addition of itraconazole for a further 6 d (Fig. 9 C). Strikingly, not only did organoids fail to recover, but organoid collapse continued to progress even in the absence of further treatment. Itraconazole treatment of PDOs also reproduced the G1 cell cycle arrest phenotype seen in other assays. Cells from PDOs treated with itraconazole for 5 d incorporated less EdU, and a higher proportion were found in G1 (Fig. 9, D and E).

Analysis of individual gene expression changes from CRC cell lines in response to itraconazole treatment revealed that although the Wnt pathway was uniformly affected in both cell lines, alterations in components of the Hh pathway were not equally represented in both lines (Data S1). Importantly, the Hh pathway defining effector genes (*Gli1-3*) were not expressed in HT55s, and only *Gli1* was detectable in SW948s, despite the same phenotype being observed in both lines. These data indicate that if the shared sensitivity of the two lines is similarly regulated, it cannot be mediated via canonical Gli effectors of the Hh pathway. To identify whether our other responsive CRC cell lines demonstrated a similar dichotomy in molecular response to itraconazole treatment, we performed RT-PCR for the Wnt target *Lgr5* and the Hh target *Gli1* (Fig. 7 E). Responsive cell lines demonstrated marked Wnt inhibition, clearly correlated with responsiveness to itraconazole. Changes in levels of *Gli1* were also detected in some lines, but bore no correlation with drug responsiveness (Fig. 7 F). Additionally, using a large, publicly available RNA expression study dataset, it was confirmed that the responsive CRC subtype (CCS1) is also characterized by high expression of Wnt components such as *Lgr5* (Fig. 7 G; Marisa et al., 2013). Hence, we hypothesized that the effects of itraconazole were mediated through inhibition of elevated Wnt signaling rather than through canonical Hh signaling inhibition. To experimentally test this, we performed small molecule inhibition of Gli function. *Gli1*-expressing SW948 cells were treated with Gant-61 (a well-validated inhibitor of both Gli1 and Gli2), and growth characteristics/expression changes were analyzed (Mazumdar et al., 2011). SW948 cells were found nonresponsive to Gant-61 treatment (Fig. 7 H). Further, despite Gant-61 treatment effectively silencing canonical Hh signaling, it had no effect on the Wnt pathway (Fig. S2 C). Next, we tested whether recombinant Shh or Ihh could rescue the itraconazole phenotype using live cell confluence measurement. Neither treatment provided rescue to the cell cycle arrest seen with itraconazole (Fig. S2 D). Suppressor of fused (SuFu) has been shown to act downstream of Smo in the Hh pathway, inhibiting Gli activation, but also enhancing nuclear β-catenin export, thereby inhibiting Wnt activity (Ding et al., 1999; Meng et al., 2001). To test whether SuFu mediates the effect of itraconazole on Wnt activity, siRNA knockdown of SuFu was performed and found to partially rescue the itraconazole-induced arrest phenotype without affecting canonical Hh pathway activation at the mRNA level (Fig. 7 I and Fig. S2, E–G). Using a T cell factor/lymphoid enhancer factor–GFP (TCF/LEF-GFP) reporter construct (to quantify Wnt pathway activity) together with SuFu knockdown, it was seen that rescue of the itraconazole phenotype is predicated on SuFu-mediated Wnt pathway activation (Fig. S2, H–J). Finally, using live cell confluence measurements, it was tested whether cyclopamine, which binds smoothened at a distinct site from itraconazole, can induce the same phenotype as itraconazole. SW948 cells were found nonresponsive to cyclopamine treatment, suggesting that itraconazole induces a different effect on smoothened than cyclopamine, which preferentially effects SuFu activity (Fig. S2 K).

Finally, RT-PCR was used to quantify the degree of Wnt inhibition generated by itraconazole treatment compared with other known preclinical Wnt inhibitors. SW948 cells were grown in 2D and treated with DMSO, itraconazole (2.5 µM), carnosic acid (60 µM), and IGC-001 (6 µM) for 3 d. Cells treated with IGC-001 immediately died and provided no material suitable for RT-PCR measurements; however, cells treated with DMSO or carnosic acid proliferated. Cells treated with itraconazole proliferated and then arrested as described earlier. RT-PCR showed significant down-regulation of Wnt target genes in itraconazole treated samples compared with carnosic acid and DMSO control (Fig. S3 A). Overall, these data confirm that the phenotype and molecular changes seen with itraconazole treatment appear driven by noncanonical Hh-driven inhibition of the Wnt pathway rather than canonical Hh pathway inhibition.

### Itraconazole treatment suppresses tumor growth using in vivo and in vitro preclinical assays

**Figure 10.**
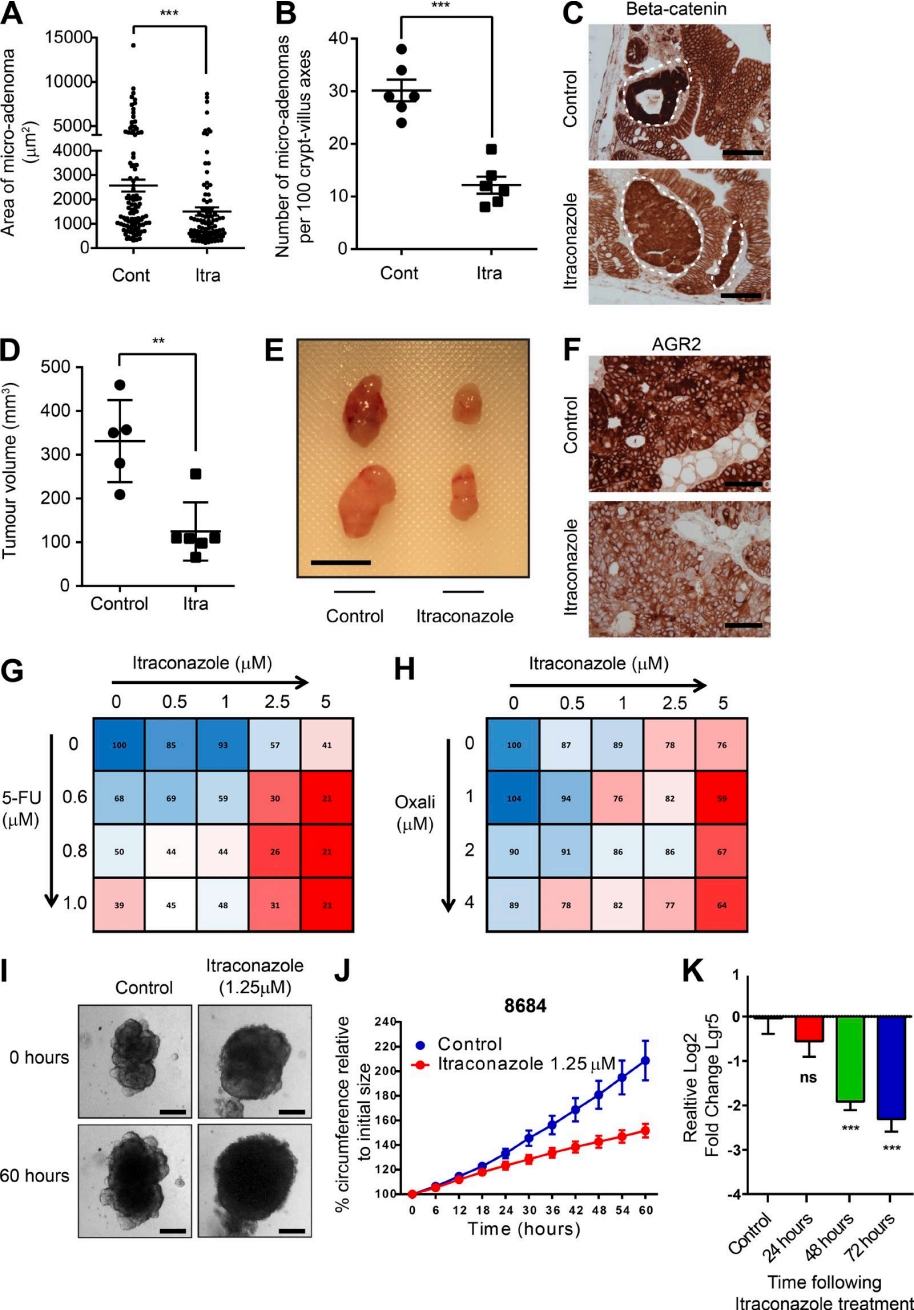
**Itraconazole retards Wnt activity and Lgr5 expression in preclinical assays. (A)** Column scatter plot showing areas of microadenomas in the presence of itraconazole or control. **(B)** Column scatter plot of the numbers of microadenomas observed per 100 crypt–villus axes in itraconazole or control treated animals. *n* = 3; mean ± SEM; ***, P < 0.001 by unpaired *t* test. **(C)** Bright field images demonstrating less-intense nuclear β-catenin staining in microadenomas of itraconazole-dosed animals compared with controls. Bars, 100 µm. **(D)** Scatter plot showing the relative tumor volumes of HT55 xenografts after oral gavage of itraconazole or water to NSG mice. *n* = 3; mean ± SD; **, P < 0.01 by unpaired *t* test. Cont, control; Itra, itraconazole. **(E)** Representative images of treated and control xenografts. Bar, 1 cm. **(F)** Bright field images of anti-AGR2 immunohistochemical staining from HT55 xenografts treated with itraconazole of control. Bars, 100 µm. **(G and H)** Matrix plots of the synergistic effects of itraconazole on oxaliplatin- and 5-FU–treated SW948 cells. Values indicate relative cellular confluence 5 d (5-FU) and 3 d (Oxali) after treatments. *n* = 3. **(I)** Representative bright field images of organoids derived from human CRC liver metastases at initial treatment date and 60 h after treatment with itraconazole. Bars, 100 µm. **(J)** Growth curves of circumference changes in patient-derived liver metastasis organoids after treatment with itraconazole. **(K)** Histogram of RT-PCR Lgr5 expression levels in patient derived liver metastasis organoids after itraconazole treatment. *n* = 12; ***, P < 0.001; ns = not significant by one-way ANOVA.

To investigate the in vivo effectiveness of itraconazole to inhibit Wnt signaling, tumor-predisposed *Lgr5-Cre^ER^_Apc^fl/fl^* mice were dosed with itraconazole or control for 10 d, followed by tamoxifen-induced Cre recombination to knockout Apc in intestinal stem cells. Mice were culled 18 d later, and small intestinal tissue was analyzed. As previously described, multiple small Wnt hyperactive microadenomas were detected throughout the small intestine in control and treated animals (van der Heijden et al., 2016). However, both the number and size of adenomas were lower in itraconazole-treated animals (Fig. 10, A and B). Anti–β-catenin staining also revealed less intense nuclear staining in adenomas from itraconazole-treated animals (Fig. 10 C). To confirm this apparent in vivo Wnt inhibitory effect, anti-GFP staining was performed to identify Wnt^High^ *Lgr5*–expressing stem cells. Anti-GFP staining was uniformly less intense or, more commonly, completely absent in the crypts of itraconazole-treated animals throughout the small intestine, confirming the in vivo Wnt inhibitory capacity of itraconazole (Fig. S4, A and B). To exclude confounding as a result of the known mosaicism in *Lgr5-GFP* expression in these mice, numbers of GFP^+^ cells were counted in crypts where there was at least one identifiable GFP^+^ cell (Fig. S4 C). Even in GFP^+^ crypts, there were dramatically fewer GFP^+^ cells in itraconazole treated mice.

To assess the preclinical utility of itraconazole for the treatment of patients with CRC, nonobese diabetic severe combined immunodeficient gamma (NSG) mice were engrafted with the Gli-negative/Hh pathway–inactive but itraconazole-responsive HT55 CRC cell line and were dosed with oral itraconazole or control (Fig. S4 D). After 3 wk of continuous dosing, tumor volumes in mice receiving itraconazole were 70% smaller than controls. (Fig. 10, D and E). Levels of the Wnt surrogate AGR2 were also found lower in itraconazole-treated animals (Fig. 10 F). To assess whether intraconazole could perturb the growth of established tumors, SW948 cells were engrafted in NSG mice as described earlier, and mice observed for 5 wk. Tumors formed in all animals. Next, mice were dosed with itraconazole or control for 10 d and then observed for further tumor growth patterns. During the treatment period, tumors in itraconazole-dosed mice failed to grow or regressed, whereas rapid growth was observed in control mice (Fig. S3 B). After cessation of treatment, tumors in control animals continued to grow in volume by 381 ± 185 mm^3^; however, there was only very limited regrowth in itraconazole treated animals of 53 ± 50 mm^3^ (Fig. S3 B).

Live cell confluence measurements were next taken to investigate for synergy between itraconazole and the most commonly prescribed chemotherapeutic drugs in CRC, 5-fluorouracil and oxaliplatin. The effects of both drugs were found augmented by itraconazole (Fig. 10, G and H). Finally, organoids grown from patient-derived CRC liver metastases and treated with itraconazole were growth retarded by 34% ± 3% (Buzzelli et al., 2018). Altered morphology was seen, as well as similar Wnt inhibitory effects, as seen in all our other assays (Fig. 10, I–K; and Fig. S3, C and D). Organoids underwent targeted hot spot sequencing, and all were found to belong to CCS1, validating the sensitivity seen (Data S1).

## Discussion

Functional cellular heterogeneity is a clinically important but neglected focus of cancer therapeutics. Although significant progress has been made in characterizing the molecular heterogeneity present between tumors, functional heterogeneity is evidently also present and will confound both targeted treatments and traditional cytotoxic therapies currently used as adjuvant treatments. For the first time, we functionally and molecularly characterize dormant tumor cells across CRC molecular subtypes, finding they are a subset of differentiated cells, capable of contextual clonogenicity. LRCs were unexpectedly found predominantly in G2 rather than G1/0, suggesting either slow cell cycle progression or G2-poised quiescence, as has recently been described (Otsuki and Brand, 2018). We find tumor LRCs to generally be inversely associated with the Wnt^Hgh^ CSC signature. These findings are distinct to our previous study in normal mouse small intestine where we found LRCs to uniformly have a similar transcriptome to Lgr5^+^ Wnt^High^ stem cells. We hypothesize that this interesting difference may be dictated by the overall increased levels of Wnt activity within all CRCs or possibly that tumor LRCs are further committed in the differentiation process than in normal epithelium. The in vitro behavior of LRCs is precisely that which was recently described for Krt20-differentiated cells, which are also highly clonogenic in culture (Shimokawa et al., 2017). We uncovered, through mouse drug screening, that itraconazole targets Wnt^High^ cycling tumor cells, but also eliminates dormant tumor cells most effectively in CCS1 tumors (Fig. S3 D). Phenotypically, itraconazole treatment induces a proliferative burst, causing all cells in responsive tumors (including LRCs and non-LRCs) to cycle briefly and then enter stable G1 arrest and senescence. Our study does not uncover the mechanism by which the initial proliferate burst in responsive lines (which also causes the temporary reentry of dormant cells into the cell cycle) is generated by itraconazole treatment, although it is striking that this behavior is reminiscent of that caused by oncogene-induced senescence (Di Micco et al., 2006). The induction of senescence, by down-regulation of Wnt signaling, seen with itraconazole treatment, has been reported in other systems, but never in the context of CRC (Ye et al., 2007; Elzi et al., 2012). The organoid collapse seen in both mouse and human organoids upon itraconazole treatment is unusual, not least in the absence of cell death or apoptosis. The maintenance of organoid structure requires the coordinated action of highly proliferative cells, and we hypothesize that the induction of arrest perturbs this process causing the organoid structure to collapse without death or apoptosis, akin to a deflating balloon.

Itraconazole has been shown in many studies to act as a bona fide smoothened inhibitor; yet, in our study we find that its Wnt inhibitory ability occurs as a consequence of noncanonical Hh signaling inhibition. As this manuscript was being revised, a recent study reported autocrine noncanonical Hh signaling as a positive regulator of Wnt signaling in CRC CSCs (Regan et al., 2017). We propose SuFu to be the master regulator linking the Hh pathway with Wnt inhibition that both we and Regan et al. describe. Several Hh inhibitors have been used in the preclinical setting in CRC, although none are in routine clinical use. Vismodegib, a small molecule smoothened inhibitor, has been used in a randomized Phase II clinical trial in treatment-naive patients with metastatic CRC (Berlin et al., 2013). Disappointingly, these results failed to show an additional benefit of the new agent to standard therapy for metastatic CRC; although, toxicity was higher in the Vismodegib arm. Other smoothened inhibitors have never been trialed in a clinical context with CRC.

The recent study from De Sousa E Melo et al. (2017) demonstrating the reliance of CRC liver metastases on Lgr5^+^ cells for their maintenance places these cells, whether inherent or induced by dedifferentiation, in addition to the Wnt pathway as a whole, as highly relevant for therapeutic targeting. Targeting of the Wnt pathway has been an important focus for drug development and is of urgent clinical importance in CRC given the pathway is commonly (∼80%) activated. Unfortunately, the Wnt pathway has been particularly difficult to pharmacologically target, and there are no drugs in routine clinical use that are effective in CRC. The Wnt inhibitor IGC-001 was markedly cytotoxic in our study, precluding comparison with itraconazole; however, itraconazole was far more efficient than carnosic acid in inhibiting the pathway. Our robust, panspecies, multiassay demonstration of Wnt signaling inhibition in response to itraconazole treatment provides a tantalizing glimpse into the potential utility of this FDA-approved drug as adjuvant treatment in CRC for targeting the Wnt pathway, *Lgr5*-expressing cells, and cell cycle heterogeneity in a molecularly defined subset of CRCs. Given the proven safety profile of itraconazole, our study provides compelling evidence for advancing itraconazole to early phase clinical trials, as well as ascertaining whether the same effect is seen with different azole antifungals and whether the drug enhances the efficacy of conventional adjuvant treatments in vivo.

## Materials and methods

### Cell culture and associated assays

All CRC cell lines were STR-genotyped and confirmed mycoplasma free. Cells were grown in standard conditions in 37°C incubators (5% CO_2_ and 5% O_2_). Media used for 2D-adherent culture were DMEM/F12 (supplemented with 10% FBS and 1% Pen/Strep) for HT55; SW1463, SW948, SW48, T84, LoVo, RKO, and DLD1, RPMI for HCT116; HCT-15 and HT29 (supplemented with 10% FBS and 1% Pen/Strep) and DMEM for SW480 (supplemented with 10% FBS and 1% Pen/Strep).

Nonadherent “stem cell culture” was performed in Corning Ultra-Low attachment flasks in serum-free media as above, supplemented by EGF (20 ng/ml), bFGF (10ng/ml), and Pen/Strep (1:100).

Live cell confluence measurements were performed using the Incucyte Live Cell Imaging System (Essen Bioscience). The confluence readings obtained were normalized to the initial confluence for each well to account for any minor differential seeding density.

CFSE labeling was performed as per manufacturer’s instructions. Cells were trypsinized, CFSE-quenched, and counted using the Vi-CELL system (Beckman Coulter). Experimental samples and unstained controls were labeled with CFSE or DMSO treatment by adding 15 µl of CFSE or DMSO to a 2 × 10^6^ cell suspension while vortexing. Samples were then incubated for 20 min at 37°C and then quenched with media containing 10% FBS and left at room temperature for 5 min. The samples were then pelleted at 1,200 rpm and resuspended in media for culture.

FACS EdU estimates were performed using the Click-iT kit as per manufacturer’s instructions in combination with PI staining to identify cell cycle position. To ascertain whether cells incorporated EdU at a late time point, cells were seeded and left to grow for 1 wk in the presence of itraconazole or control. Cells were then labeled with EdU (10 µM) for 2 h, then trypsinized, fixed, and labeled with PI. FACS analysis was then performed for the proportion of EdU-positive cells. To ascertain the cell cycle destiny of cells during the proliferative burst and subsequent arrest, cells were seeded and left to grow for 2 d in itraconazole or control. Cells were then labeled with EdU for a further 24 h and then trypsinized, fixed, and stained with PI. FACS analysis was then performed to quantify the proportion of EdU-positive cells in the G1 phase of the cell cycle.

For SW948 cells, 6 × 10^4^ cells were transfected using Lipofectamine 2000 or 3000 (Life Technologies) in 48-well culture plate. Cells were analyzed for mRNA levels 2 and 6 d after transfection. The siRNAs used were obtained from Dharmacon: siGENOME Control Pool Nontargeting no. 1 (D-001206-13-05) and siGENOME SMART pool Human SuFu (M-015382-00).

A Qiagen Cignal TCF/LEF-GFP reporter assay was used as per manufacturer’s protocol to quantify Wnt pathway activation. Lipofectamine 3000 was used as the transfection agent. Positive and negative controls were used to quantify transfection efficiencies, unexpected effects, and to normalize data. FACS analysis of transfected cells included single cell and PI (live/dead) gating to control for the altered cell growth seen with different treatments.

Annexin V staining was performed using as per manufacturers protocol using AF350-conjugated Annexin V (Thermo Fisher Scientific).

### Mathematical modeling of CFSE dilution

To identify whether our population was composed of a bulk population together with one possessing different cycling properties, we developed a mathematical model of cell division, similar to that described in León et al. (2004). Each cell within the population will double, entering the next generation at time *t* according to the probability of cellular divisions of bulk population *K_bulk_* (*t*) and the slowly dividing population *K­_slow_* (*t*).

#### Cell doubling

The probability that a cell from either population has divided *n* times at time *t* can be written as

$$L_{n}\left(t\right)=\int_{0}^{t}K\left(t-\tau \right)L_{n-1}\left(\tau \right)d\tau ,$$ (1)

where *K* is a probability of cellular division with time and *L_n_* is the probability the cell population divides *n* times at time *t*, given an initial uniform division probability *L*_0_. Therefore, the probability of dividing at least *n* times is

$$H_{n}\left(t\right)=\int_{0}^{t}L_{n}\left(\tau \right)\;d\tau .$$ (2)

The number of cells in generation *n*, accounting for those lost to the next generation and starting with *N*_0_, is

$$N_{n}\left(t\right)=2^{n}N_{0}\;\left[H_{n}\left(t\right)-H_{n+1}\left(t\right)\right].$$ (3)

#### Cell division times

The intrinsic distribution of cell division times (*K*) is taken to be an inverse gamma distribution proposed in León et al. (2004):

$$K\left(t,t^{bulk\;or\;slow}\right)=\left\{ \begin{array}{c} 0,\;t<t^{b,s},\\ \frac{1}{\sigma \Gamma \left(\lambda \right)}{\left(\frac{t-t^{b,s}}{\sigma }\right)}^{\lambda -1}E^{-\left(\frac{t-t^{b,s}}{\sigma }\right)},\;t\geq t^{b,s}. \end{array}\right.$$ (4)

This distribution has a minimum cycle time *t^bulk or slow^* and shape parameters *σ* and *λ*, which control the broadness of the tail. These later parameters provide the potential for increasing the likelihood of cells with long cycle times.

#### CFSE intensities

The normalized intensity for each population can then be calculated using the initial intensity *I*_0_(*i*) as

$$I_{t}\left(i\right)=\sum_{n=1}^{\infty }\frac{I_{0}\left(i\right)}{2^{n-1}}N_{n}\left(t\right)/\int_{0}^{\infty }\sum_{n=1}^{\infty }\frac{I_{0}\left(i\right)}{2^{n-1}}N_{n}\left(t\right)\;di.$$ (5)

Finally, the overall model intensity is calculated as the weighted mixture of bulk and slow populations as

$$I_{t}^{total}\left(i\right)=\left(1-\alpha \right)I_{t}^{bulk}\left(i\right)+\alpha I_{t}^{slow}\left(i\right).$$ (6)

#### Parameter estimation

The best fit for the predicted model intensity $I_{t}^{total}$ to that observed in the CFSE data at day 6 is found through least squares minimization in terms of our model parameter (*t^bulk^*, *σ^bulk^*, *λ^bulk^*, *t^slow,^ σ^slow^*, *λ^slow^*, *α*) using Matlab’s fminsearch algorithm.

### Mouse models

*Ah-H2B-YFP*, *Apc^1322T^, Lgr5-Cre^ER^_Apc^fl/fl^* and *NSG* mice have previously been described (Moser et al., 1990; Pollard et al., 2009; Buczacki et al., 2013; van der Heijden et al., 2016). Mice were bred and housed according to UK Home Office guidelines. All animal experimentation was performed in accordance with the Animal (Scientific Procedures) Act 1986, the European Union Directive 86/609, and with local (CRUK CI, University of Cambridge) ethics committee approval.

Tumor xenograft volume (H × W × D/2) was calculated using callipers in three-dimensions after culling of the animals and dissection of the tumor from the subcutaneous tissue. Mice were sex, litter, and age matched.

### Mouse tumor disaggregation and flow cytometry

Mice with intestinal tumors were sacrificed and dissected when displaying signs of tumor burden. Sections of PBS-flushed small intestine were opened and pinned out on silicone plates under cold 2% FBS/PBS. Visible tumors were dissected free from the tissue and minced with scissors. Minced tumor material was resuspended in 10 ml of prewarmed HBSS (-Ca^2+^/-Mg^2+^) supplemented with 10 µM EDTA and 10 mM NaOH and incubated in a water bath at 37°C for 10 min with regular agitation. The sample was then allowed to settle, and the supernatant aspirated and combined with 20 ml of ice-cold 2% PBS/FBS. The remaining sample was resuspended in a further 10 ml of HBSS for a further 10 min, and the process was repeated one final time. All samples were then pooled and spun down at 1,200 rpm for 5 min. After washing in 2% FBS/PBS, the samples were resuspended in 2 ml of Dispase (5 mg/ml) and 200–500 µl of DNase and incubated with agitation for 7 min. The sample was then filtered through a 100-µm mesh to gain a reliable single cell preparation. The single cell preparation was then washed in 2% FBS/PBS.

### Mouse adenoma organoid culture

Mouse adenomas were cultured as per previously described (Sato et al., 2011b). In brief, single-cell preparations of tumor cells were plated into a 96-well plate. Each well of the plate was precoated with 40 µl of 50:50 Advanced DMEM/F12 (ADF)/Matrigel mix. Cells were resuspended in ADF-supplemented 2% Matrigel (2%), N2 (1:100), B27 (1:50), Pen/Strep (1:100), Rocki (10 µM), and EGF (50 ng/ml) at a concentration of ∼8 × 10^5^/ml. 125 µl of the ADF/Cell mix was then pipetted on top of the Matrigel.

### 3D mouse adenoma organoid drug screen

YFP-1322 mice were housed until a tumor phenotype developed, typically anemia at around 90 d. The mice were then sacrificed and small intestinal tumors processed as above (see Mouse tumor disaggregation and flow cytometry), with 24 wells being used for each drug tested. Cells were seeded and left to form organoids over the following 4 d. On day 5, organoid YFP expression was induced with 2.9 nM βNF dissolved in DMSO for 24 h. The following day, the βNF/media was removed, and media were replaced including drugs at three concentrations or carrier control (equal volume), in replicates of six (see Data S1). Drugs were selected based on literature review of commercially available small molecules or recombinant proteins that would agonize or antagonize pathways known to be involved in the control of intestinal stem cell homeostasis or implicated in colorectal cancer development. The lowest concentration used was the stated IC50, the middle concentration was 2.5× IC50, and the highest was 5× IC50. At the end of the experiment (day 9), the entire plate was imaged using the GelCount (Oxford Optronix), and organoid number and size were calculated using the manufacturer’s software after optimization. After imaging, half the wells underwent RNA extraction using the RNeasy Micro Plus kit as per manufacturer’s instructions. The remaining 12 wells were processed for FACS analysis. In brief, after aspiration of media, 100 µl of matrigel recovery solution was applied for 45 min on ice. Next, 100 µl Accutase was added at room temperature with regular trituration to enable organoids to disaggregate to a single cell preparation. Flow cytometric analysis for YFP expression and viability (DAPI or PI) was then performed. Expression data are presented from “High”-treated samples only.

### Human tumor organoid isolation and culture

#### Primary CRC PDO

Colonic tissues were obtained from hospitals around the UK as part of the Human Cancer Models Initiative with informed consent, and the study was approved by the London–Camden & Kings Cross Research Ethics Committee. Colon tumor samples were taken from resected colons, and the isolation of tumor epithelium was performed as previously described (Sato et al., 2011a). Tumor samples underwent multiple washes with PBS before being minced into small pieces using a scalpel and incubated with collagenase II (10 mg/ml) for 1–2 h at 37°C. After incubation, the mixture was filtered through a 70-µm cell strainer to remove large undigested fragments. The cell suspension was centrifuged at 800 *g* for 2 min. The cell pellet was resuspended in PBS and centrifugation repeated. This procedure was repeated twice to remove debris and collagenase.

The isolated cells were resuspended in 12 mg/ml basement membrane matrix (Cultrex BME RGF type 2, Amsbio, BME-2) supplemented with complete media and plated as 10- to 15-µl droplets in a 6-well plate. After allowing the BME-2 to polymerize, complete media was added and the cells left at 37°C.

#### Complete media

AdDMEM/F12 medium supplemented with Hepes (1×; Invitrogen), Glutamax (1×; Invitrogen), penicillin/streptomycin (1×; Invitrogen), B27 (1×; Invitrogen), Primocin (1 mg/ml; InvivoGen), N-acetyl-l-cysteine (1 Mm; Sigma), RSPO1-conditioned medium (20% vol/vol; cells provided by C. Kuo [Stanford University School of Medicine, Stanford, CA]). A cell line is also available from Trevigen), recombinant Noggin protein (0.1 µg/ml; Peprotech), EGF (50 ng/ml; Peprotech), Nicotinamide (10 Mm; Sigma), SB202190 (10 µM; Stem Cell Technologies), and A83-01 (0.5 µM; Tocris).

Organoid culture medium was refreshed twice a week. To passage the organoids, BME-2 was disassociated by pipetting. The organoids were collected into a falcon tube, and TrypLE (Invitrogen) was added before being incubated at 37°C for ∼5 min. A vigorous manual shake would ensue before the suspension was centrifuged at 800 *g* for 2 min. The remaining cell pellet was resuspended in 12 mg/ml BME-2 supplemented with complete media and plated as 10- to 15-µl droplets in a 6-well plate. After allowing the BME-2 to polymerize, complete media was added and the cells left at 37°C.

#### Liver metastases PDOs

Tissue was acquired from the Oxford Radcliffe Biobank with informed consent. The study was approved by the South Central-Oxford C Research Ethics Committee. Organoids were derived from human liver metastases of colorectal cancer using rapid isolation as previously described (Ashley et al., 2014; Buzzelli et al., 2018). Organoids were grown in DMEM/F12 + GlutaMAX containing StemPro, ROCK inhibitor, R-Spondin-1 (RSPO-1), Noggin, WNT3A, EGF, Insulin-like Growth Factor 1 (IGF-1), Fibroblast Growth Factor 10 (FGF-10), Fibroblast Growth Factor basic (FGF-β), and Endothelin 3 (ET3). Expression of colonic markers were assessed in organoid cultures and original tumor specimens. All organoids showed similar expression of colonic markers as their respective tumor specimen confirming their origin (Buzzelli et al., 2018). Organoids were passaged 2 d before itraconazole treatment. For itraconazole treatment, organoids were given fresh media containing 1.25, 2.5, or 5 µM itraconazole or untreated media. Time-lapse images were captured every 3 h for 60 h on a Nikon Eclipse Ti-E inverted microscope system. Images were converted to TIF files and the area of organoids were measured using in-house software written in MATLAB R2015b software.

### SA-β-GAL staining

Cells were washed with PBS and fixed with 0.5% glutaraldehyde in PBS for 15 min at room temperature. Cells were then washed with 1 mM MgCl_2_ in PBS, pH 6.0. X-Gal staining solution was incubated overnight at 37°C. Cells were then washed in PBS and visualized on a bright field tissue culture microscope.

X-Gal staining solution: 1× X-Gal and 1× KC solution in MgCl2/PBS. KC solution: 0.32g K_3_Fe(CN)_6_, 1.05g K_4_Fe(CN)_6_ × 3H_2_0, and 25 ml PBS, pH 6.0.

### RT-PCR

SYBR green RT-PCR was performed under standard conditions using a Rotorgene (RG3000; Corbett Research) or a QuantStudio 12K Real-Time Flex System (Life Technologies). Custom primers were validated before use using standard SYBR green qRT-PCR and agarose gel electrophoresis of PCR products. Samples were normalized to housekeeping genes *β-actin* or *β2 microglobulin*.

TaqMan RT-PCR was performed under standard conditions as above. Samples were normalized to housekeeping genes ribosomal protein *L19* and/or *β2 microglobulin*. All TaqMan probes underwent initial efficiency validation using standard curve analysis.

#### Human metastatic organoid RT-PCR

RNA was harvested using RNeasy purification kit (Qiagen). RNA was reverse transcribed using Moloney mouse leukemia virus reverse transcription (Promega) primed with oligo (dT). Quantitative RT-PCR primers were designed using PRIMER EXPRESS (Applied Biosystems). SYBR green chemistry was used with rL32 as the internal reference gene. The conditions were 95°C for 10 min, 40 cycles of 95°C for 15 s, and 60°C for 15 s (Mx3005P; Stratagene). Results were analyzed using sequence detector software, relative fold differences were determined using the ΔΔCt method.

### Drug synergism assay and analysis

HT55 and SW948 cells were seeded at concentration of 10^5^ cells per ml in a 96-well plate. Cells were treated with a range of itraconazole (0–5 µM) and 5-fluorouracil (5-FU; Sigma; 0–0.8 µM) or Oxaliplatin (Sigma; 0–4 µM) concentrations in quadruplicate for each drug combination. All reagents were dissolved in dimethyl sulfoxide (Sigma). Cell growth over time was measured using an Incucyte ZOOM instrument. Data were analyzed after 3 (Oxaliplatin) and 5 d (5-FU) of treatment. Data were normalized to lowest seeding value. Final growth values for each treatment were calculated as percentage of the vehicle control value.

### RNA sequencing and bioinformatic analysis

RNA was extracted using the RNAeasy Micro Plus kit (Qiagen) and quantified using the Qubit RNA Assay kit (Thermo Fisher Scientific). RNA quality was assessed using the Agilent Bioanalyzer system as per manufacturer’s instructions. After normalization and sample randomization, Truseq library (Illumina) preparation was performed at the CRUK CI genomics facility and subsequent single-end, 50-bp sequencing using the HiSeq system (Illumina). After human genome alignment (hg19), read counts were normalized, and differential expression was tested using the DEseq protocol (Mortazavi et al., 2008; Anders and Huber, 2010). Data were deposited to the GEO database under accession no. GSE114014.

GO pathway analysis was performed using the online tool from GeneGO Metacore. Further bioinformatics analysis was performed using the GENE-E and Morpheus platforms (Broad Institute) on the Cancer Cell Line Encyclopedia database and the expression database of Marisa et al. (2013). GSEA was performed using the Broad GSEA tool. Genes were preranked based on fold change.

All experiments were performed in biological quadruplicate.

For human T-LRC (CFSE^+^) characterizations, as a control experiment to identify off-target effects of the CFSE dye and culture artifacts, bulk populations from DLD1 cells at days 1 and 6 after seeding, both with and without CFSE labeling, were included in the RNAseq analysis. Principal component analysis (PCA) of these control experiments showed that of the transcriptomic differences seen in this experiment, ∼97% were generated by time in culture, and <1% were attributable to the dye.

#### PDO targeted gene sequencing

For targeted sequencing, we used a custom cRNA bait set (Agilent SureSelect and WTSI v4 Panel) to enrich for all coding exons of 279 cancer genes. Short insert libraries (150 bp) were prepared and sequenced on the Illumina HiSeq 4000 using 75-bp–end sequencing as per Illumina’s protocol. The mean sequence coverage was ∼800× for the tumor samples. Sequencing reads were aligned to the reference human genome (GRCh37d5) using BWA-MEM (v0.7.15; Li, 2013), CaVEMan (v1.11.2) was used for calling substitutions (Jones et al., 2016), and Pindel (v2.2.2) for small insertions and deletions (Raine et al., 2015). Matched blood sample to remove germline mutations and somatic mutations were screened against a list of known cancer mutations to flag potential drivers.

### Statistical analysis

Statistical analysis was performed in Excel (Microsoft) and Prism v6 (GraphPad). All data were assessed for normality using the D’Agostino and Pearson test. Significance of parametric data were tested using a two-tailed Student’s *t* test. For nonparametric data, the Mann Whitney *U* test was used. For multiple group comparisons, ANOVA testing was performed. Raw experimental data are presented in Data S1.

### Primers and probes

#### Mouse TaqMan probes

LP19, Mm02601633_g1; β2-MG, Mm00437762_m1; EphB2, Mm01181021_m1; Axin2, Mm00443610_m1; Msx1, Mm00440330_m1; Id1, Mm00775963_g1, p21, Mm04205640_g1, Hes1, Mm01342805_m1; Cox-2, Mm03294838_g1; CyclinD1, Mm00432359_m1; Gli1, Mm00494654_m1; Ptch1, Mm00436026_m1; Lysozyme, Mm00657323_m1; MMP7, Mm00487724_m1; Muc2, Mm01276696_m1; Dll1, Mm01279269_m1; Atoh1, Mm00476035_s1; ChgA, Mm00514341_m1; Sox9, Mm00448840_m1; CDX1, Mm00438172_m1; Villin, Mm00494146_m1; Lgr5, Mm00438890_m1; Bmi1, Mm03053308_g1; PW1, Mm01337379_m1; DCAMKL-1, Mm00444950_m1; Notch1, Mm00435249_m1, CD133, Mm00477115_m1; and CD44, Mm01277163_m1.

#### Human TaqMan probes

Lgr5, Hs00173664_m1; Smo, Hs01090242_m1; Ptch1, Hs00181117_m1; Shh, Hs00179843_m1; β-actin, Hs01060665_g1; Axin2, Hs00610344_m1; RPL19, Hs02338565_gH; EphB2, Hs00362096_m1; Cldn1, Hs00221623_m1; Sox9, Hs00165814_m1; CDX2, Hs01078080_m1; Muc2, Hs03005103_g1; and PTK7, Hs00897151_m1.

#### Human primers

*rL32* forward, 5′-CATCTCCTTCTCGGCATCA-3′; *rL32* reverse, 5′-ACCCTGTTGTCAATGCCTC-3′; *Lgr5* forward, 5′-AACAGTCCTGTGACTCAACTCAAG-3′; *Lgr5* reverse, 5′-TTAGAGACATGGGACAAATGCCAC-3′; *Shh* forward, 5′-TTATCCCCAATGTGGCCGAG-3′; *Shh* reverse, 5′-TACACCTCTGAGTCATCAGCC-3′; *Ihh* forward, 5′-TCCGTCAAGTCCGAGCAC-3′; *Ihh* reverse, 5′-CTCGATGACCTGGAAGGCTC-3′.

### Drug screen compounds and other reagents

HGF (R&D), Dkk1 (R&D), Wif1 (R&D), Draxin (R&D), sFRP1 (R&D), sFRP5 (R&D), BMP4 (R&D), Gremlin (R&D), Jagged 1 (Anaspec), BMP3 (R&D), BMP7 (R&D), Dll1 (R&D), Dll4 (R&D), DAPT (Sigma), DAPT-GSI (Sigma), TGF-α (R&D), Lrig1 (R&D), Shh (R&D), Ihh (R&D), Itraconazole (Sigma), Gant-61 (Tocris Bioscience), Calcitriol/Vit D (Sigma), Rapamycin (LC Laboratories), ICG-001 (Tocris), and Carnosic Acid (Sigma; also see Data S1)

Xenograft and in vivo itraconazole dosing experiments: Beacon Pharmaceuticals (10 mg/ml).

Antibodies for IHC and FACS: AGR2 (Atlas, HPA007912), GDF15 (Atlas, HPA011191), β-catenin (BD Biosciences, 610154), and PTK7 (clone 188B; Miltenyi).

### Online supplemental information

Fig. S1 shows the mathematical modeling used to identify the CFSE^High^ dormant population, summary data from RNAseq characterizations of LRCs, and the plasticity seen in LRC populations. Fig. S2 demonstrates itraconazole mediates its effect through noncanonical Hh signaling mediated by SuFu. Fig. S3 summaries shows additional preclinical validation of the effect of itraconazole and a summary diagram explaining its effectiveness. Fig. S4 shows the effect of itraconazole in *Lgr5-Cre^ER^_Apc^fl/fl^* mice. Data S1 contains summary RNAseq DE tables, phenotype signatures, additional drug data, PDO sequencing data, and raw experimental data.
